# Common scents? A review of potentially shared chemical signals in the order Carnivora

**DOI:** 10.1093/chemse/bjaf019

**Published:** 2025-06-09

**Authors:** Holly Root-Gutteridge, Neil de Kock, Madeleine Young, Andrew C Gill, Jake A Penny, Thomas W Pike, Daniel S Mills

**Affiliations:** School of Life & Environmental Sciences, University of Lincoln, Lincoln, Lincolnshire, United Kingdom; School of Life & Environmental Sciences, University of Lincoln, Lincoln, Lincolnshire, United Kingdom; School of Life & Environmental Sciences, University of Lincoln, Lincoln, Lincolnshire, United Kingdom; School of Life & Environmental Sciences, University of Lincoln, Lincoln, Lincolnshire, United Kingdom; School of Life & Environmental Sciences, University of Lincoln, Lincoln, Lincolnshire, United Kingdom; School of Life & Environmental Sciences, University of Lincoln, Lincoln, Lincolnshire, United Kingdom; School of Life & Environmental Sciences, University of Lincoln, Lincoln, Lincolnshire, United Kingdom

**Keywords:** chemical signals, carnivore, mammal, odor, pheromone

## Abstract

Many animals transmit information in the form of chemical signals to modify behavior or induce physiological change in receivers. For some taxa, such as species in the order Carnivora, chemical signals are known to provide a critical mode of communication, although they are still poorly understood compared to other signal modalities. Here, we review the literature to identify and categorize potential chemical signals within the Carnivora with a view to determining commonalities based on sex, taxon, and function. Data were drawn from 112 publications, dating from 1896 to 2021. Of the 1,532 discrete chemicals identified, 169 were detected in > 5 species, with 58 found in ≥ 10 species. However, multiple different names were often used to report the same compound, reducing the transparency of the literature. Two hundred and fifty-two chemicals were identified as biomarkers, i.e. associated with specific behavioral contexts (dominance hierarchy, appeasement, agonistic, etc.) or specific demographic traits (age, sex, etc.). Few studies established a causal link between these biomarkers and behavioral or physiological changes, so only a few could be definitively described as behaviorally functional bioactive signals. We found high variability concerning which species, chemicals, and sources were represented in the literature, which could potentially lead to a perceptual bias in the relative importance of certain chemicals. Finally, we propose a method for standardized reporting of chemical signals and suggest that future work should focus on a more consistent expansion of the range of species, products, and chemical types analyzed so that the phylogenetic relationship of chemical signals across taxa can be determined.

Animals communicate by providing information, in the form of a variety of different signals, to other individuals; these other individuals can then incorporate the information gained into their own decision making (Bradbury and Vehrencamp 1998). The signals take many forms, from the familiar, such as the songs of blackbirds (*Turdus merula*) or the wagging tail of a pet dog (*Canis familiaris*), to signals that require specialist equipment for humans to detect, such as the low-volume ultrasonic courtship sounds of male moths (*Ostrinia furnacalis*) (Nakano et al. 2008) or the UV-reflectant patterns of the dewlap of Puerto Rican anole lizards (*Anolis pulchellus*) (Fleishman et al. 1993). Chemical signals can also encode a wide variety of information: from species identity (Greene et al. 2016), sex (Zhang et al. 2005, 2008), age (Kean et al. 2011), and individuality (Brown and Johnston 1983; Hagey and MacDonald 2003), to pair bond (Jordan et al. 2015)  or group membership (Noonan et al. 2019). However, specific identification of the chemicals involved requires specialist equipment, e.g. mass spectrometry (Albone et al. 1974). With a growing range of chemical analytical techniques available, it has become apparent that the physical basis of a chemical signal may be described at a variety of levels, ranging from the general medium used to convey the message (e.g. urine) to the chemical class (where a class such as “fatty acid” encompassing many chemical species) to a specific molecular definition based on the physical shape of the molecules, such as a specific isomeric form of importance, or the quantitative levels of specific chemicals in a complex mixture (Fig. 1). What is labeled as the potential signal will, therefore, reflect the level of analysis undertake, and it often remains unknown at what level molecular definition is important, with advances in chemoreceptor molecular biology highlighting not only the importance of the shape of the molecular binding site but also the pattern of binding across multiple sites within the receptor (Genva et al. 2019). The potential importance of molecular combinations adds another level of complexity to the process of signal definition. Thus, whilst it is recognized that chemical signals can provide valuable insights into animal behavior and communication, this area is a potential minefield for those not familiar with the related chemistry.

**Fig. 1.**
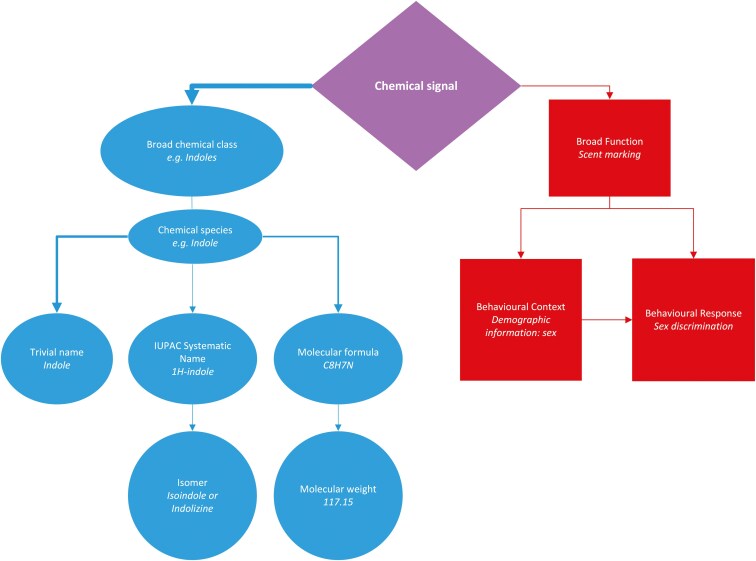
Representation of the levels of analysis of chemical signals for a) chemical identification and b) association with function, with the example of an indole given in italics. The process may start with the analysis of urine as the general source of the chemical signal. Indole, a potentially behaviorally important aromatic organic compound has many variants. The definition of the chemical signal may be refined at one or more of the levels as illustrated, from broad chemical group to isomer. Any of these may be associated with testing of a behavioral context and/ or function. Commonly, the trivial name is used for the analyte (i.e. “indole”), less commonly, are signals identified at the isomeric level (i.e. “indolizine” or “isoindole”) or by reference to molecular weight. Indole itself can also be used a general term, and as a descriptor of a class of chemicals. Likewise, the general function of a signal (e.g. territorial marks made with urine) may be identified, but the specific behavioral response to the signal is less commonly tested. Thus, both the molecular characterization and the behavioral effects can vary in their specificity. For indole, a specific behavioral response has not been linked to specific isomers and may not exist.

As already noted, a major challenge to studying a species’ chemical signaling is that the criteria and terminology which are used to refer to these signals varies widely across reports. This is not merely a matter of semantics as there is an important difference between an analyte, which is merely present in a potentially important medium, and a semiochemical, which encodes biologically relevant information. The latter should be shown to produce a behavioral or neurobiological response. Chemical analysis of scent systems is complicated by the multitude of contributing factors that need to be considered in the analytical process: such as sample origin and the complexity of the sample matrix through to issues such as potential temporal sample degradation the longer chemical analysis is from sample collection. These are common challenges faced by all chemical analyses of biological samples which must be overcome to ensure reliable characterization of candidate compounds. However, chemical scent analyses must further show that candidate compounds elicit a biological response. As such this review will be making a distinction between biomarker compounds (analytes present and which may differ between groups or populations but do not necessarily mediate behavior) and bioactive scent compounds (analytes present and which may differ between groups or populations and mediate behavior).

In addition, there is an ongoing nomenclature issue with the same chemical having multiple names, or descriptions which may depend on the specific context (Fig. 1); this may range from the basic formula or common name to the exact isomer (e.g. even a simple molecule like propanol, basic chemical formula C_3_H_8_O, has three isomers spanning 2 chemical families: alcohols propan-1-ol with structural formula CH_3_CH_2_CH_2_OH, and propan-2-ol with structural formula CH_3_CH(OH)CH_3_—common name: isopropanol or isopropyl alcohol; as well as the ether methoxyethane CH_3_OCH_2_CH_3_). As compounds get bigger the complexity and potential arrangements increase exponentially, thus basic atomic compositions become increasingly inadequate as they can be applied to myriad compounds depending on the size. This significantly complicates designing suitable studies capable of ascertaining the specific chemical identity of compounds involved in scent / signaling systems. In such cases, it is important to recognize that neither biology nor chemistry can answer all the questions, biological experiments cannot determine chemical identity and structure, whilst chemical analyses cannot determine biological response. Thus, the two must operate in the form of a mutual feedback system, in which experimental outcomes inform one another on how to proceed with future analyses. A simple example may include, biological response is noted to be chemically mediated, analytical methods may separate and fractionate the chemical analytes from collected samples for use in a future scent panel, the outcomes of which may then inform what broad components of the analytical separation are to be focused on. Such projects are far more complex, with many nuances unique to the study system in question that need to be considered. A few examples from the literature that highlight the effective coupling of biological assays with analytical analysis for characterizing bioactive compounds include Payne et al. (2018), Bohman et al. (2014), and Riffell et al. (2002).

Further complicating scent analysis is that many chemical signaling mechanisms may involve the requirement of multiple compounds at the same time to elicit a biological response and that quite different molecules may have similar effects. For example, it has been demonstrated in the 2004 Nobel Prize work by Linda Buck and Richard Axel that in the case of olfactory receptors, analytes of different sizes that share similar functional groups are able to bind with the same or several other receptors (Buck and Axel 1991). Given many biological scent systems remain severely understudied and chemical information on scent compounds is sparse or non-existent in most cases, investigations into this system may often start by assessing whether any of the most prevalent biomarkers demonstrate bioactive properties. Future work could and should explore the extent to which receptors bind to different molecules, how the brain circuits are stimulated by molecules both in isolation and in combination, and if these responses differ across taxa. This is addressed further elsewhere by Kurian et al. (2021) and Menini (2009). By contrast, this review seeks to highlight the importance of coordinating the biological and chemical approaches to the investigation of complex signaling mechanisms (thus a comprehensive review on coordinating tandem bio-assay and analytical analysis is outside the scope of the current review).

Potentially biologically important analytes have historically been categorized under a wide range of terms, including pheromone, allelomone, kairomone, allomone, synomone, chemo-signal, and odorant (Karlson and Lüscher 1959; Whittaker and Feeny 1971; Touhara and Vosshall 2009; Wyatt 2010; Nielsen et al. 2015). Some of these terms imply specific characteristics, such as the way in which the chemical is functionally bioactive, while others refer more to chemical properties. As we wish to avoid the implications associated with the use of such terms and to remain consistent, we have chosen to use the general term “chemical signal” throughout this review. Where we need to distinguish those analytes that are present but do not have an established function from those with a known behavioral function, we will use the terms “biomarker” vs “bioactive signal.” Wherever possible, we will also use standardized molecular terminology rather than trivial names (e.g. acetone would be referred to by its systematic name: 2-propanone). Furthermore, we shall use the term “sample matrices” to describe the various media in which the chemicals can be present. Thus, “sample matrix” refers to the chemical mixture in which analytes of interest (chemical scent compounds) are present. The “sample matrix” is often derived from glandular secretions and/or waste excretions.

This review focuses on the chemicals found in the Order Carnivora, their identity as biomarkers, and their potential as bioactive signals. Since the first studies of skunks in the 1890s (*Mephitis mephitica*) (Aldrich 1896; Aldrich and Jones 1897), there has been a considerable amount of research into Carnivora’s chemical signals, with developments reflecting changes in chemical analytical methods (Fig. 2). As the order Carnivora contains more than 290 species across 12 Families, i.e. Canidae, Felidae, Herpestidae, Hyaenidae, Mephitidae, Mustelidae, Odobenidae, Otarridae, Phocidae, Procyonidae, Viverridae, and Ursidae (IUCN 2020), and these taxa vary widely in size, habitat, and abundance (Nowak 1999), it is likely that their chemical signal use and production varies according to social requirements and environment. Furthermore, there are shared behaviors across taxa that make chemical signals useful, e.g. many Carnivorans are territorial and demarcate their territories with scent marks to make their presence known, using products including urine, feces, anal gland secretions, and/or marking fluid (Sillero-Zubiri and Macdonald 1998; Burger et al. 2008; King et al. 2016), thus chemical signaling is clearly important to many species. This makes them a potentially rich area for research into both chemical communication as well as the associated issues related to the identification of signals. However, research is often species- or family-specific, and less effort has been applied to identifying potential commonalities in both the presence and function of chemical signals across the Order. Here, we draw together these disparate approaches to examine the role of chemical signals in the Order Carnivora in general.

**Fig. 2.**
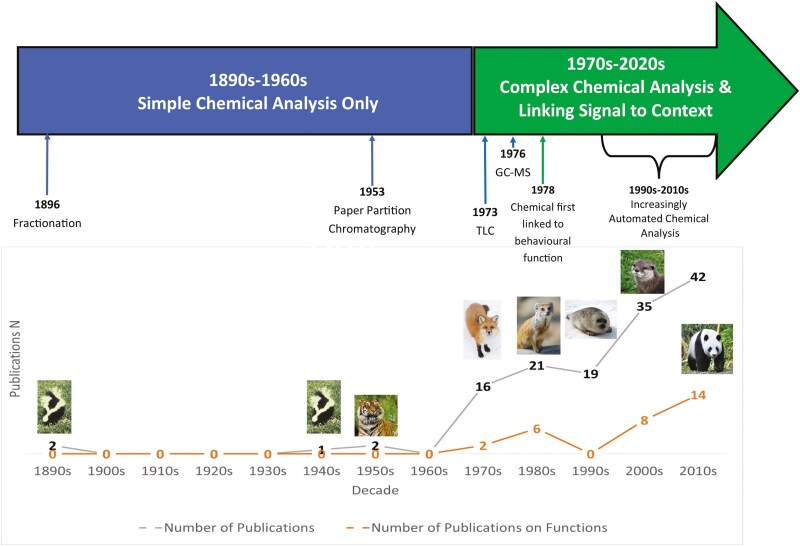
Timeline of the rate of reporting of chemical signal analysis from its origins in 1896 through to 2010s. (TLC = thin layer chromatography, GC-MS = gas chromatography-mass spectrometry.) Each picture is a carnivore species included in the chemical analysis for the first time in that decade. While the signaler may produce a signal to target a particular individual, they do not control who receives it. Thus, their unintended audience may include such diverse recipients as potential mates, competitors, predators, prey, and scientific researchers.

Furthermore, the presence of information does not necessarily require the receiver to respond to it, thus signals may be present but not acted upon in all circumstances, even if they have a bioactive property. This presents a challenge to identifying which chemicals carry semiotic information. Due to the difficulty of analyzing the myriad of potential behavioral functions, signal analysis often focuses on characterizing the chemical composition of the sample matrix and their variation between groups, i.e. establishing which are biomarkers, without associating its content with functions, (role as a bioactive signal), but both are required to define a signal (Fig. 1).

Thus, here we focus on the current literature concerning *potential* bioactive chemical signals in Carnivora, the identity of chemicals present in different products (e.g. urine, anal sac secretions, feces, etc.), the analytical methods used, the products sampled, and the commonalities observed across species and taxa.

In undertaking this review, our primary aims were to:

- Collate information on the known chemicals from literature sources, which have been reported to be present in different species in the order Carnivora and have the potential to be chemical signals, in order to identify data gaps in the choice of taxa and analysis.
- Identify any commonalities between taxa in the presence of these potential chemical signals in terms of
  - General analyte presence and their potential as biomarkers
  - Functions and their chemical analytes, their potential as bioactive signals
- Highlight issues relating to the diverse levels of chemical description used, so that we can produce recommendations for the standardized reporting of potential chemical signals in the scientific literature and especially behavioral science.
- Present a case for the use of chemical signals as phylogenetic tools to further explore the evolutionary history of the Carnivora.

## Methods

### Study eligibility criteria for inclusion in review

Search years ranged from 1880 to 2021, with the earliest identified publications from the 1890s (Aldrich 1896; Aldrich and Jones 1897). Only English-language publications were considered. Data from publications in peer-reviewed journals, reports, patents, or academic theses were considered eligible.

### Information sources

Google Scholar (years: 1890 to 2021) and Web of Science (years: 1970 to 2021) were searched for relevant sources with the final date of 28 February 2021. The database Pherobase (www.pherobase.com), which contains listings of pheromones across many taxa, was also searched for each species.

### Search

The peer-reviewed literature was systematically searched using the keywords “pheromone,” “carnivora,” “semiochemical,” “chemosignal,” “urine,” “feces”/“feces,” “scent-marking,” “anal gland,” “anal sac,” “chemical signaling”/“chemical signaling,” “scent,” genus names, and the various species of interest names (e.g. “fox,” “dog,” “cat,” “wolf,” “skunk,” etc.). Publications of interest were also identified from the bibliographies of reviewed literature. This allowed the inclusion of relevant reports that did not contain the specific keywords chosen or that were not readily discoverable in the databases.

### Study selection

One hundred and twelve studies that reported potential chemical signals were analyzed, the key pieces of information were extracted, and the relevant methods/results were entered into a bespoke database (see below). Only studies that included information on species in the order Carnivora were included in the review. Data were compiled from the existing sources in peer-reviewed literature and theses.

### Variation in the presentation of data in individual studies and across studies

To assess how different authors had presented data both within individual species and in their selection of species, we explored (i) the detail of data that had been presented and (ii) the limitations of the analytical method(s) used. Across studies, variation was judged by (i) the type of analytical methods used, with the assumption that, for example, paper partition chromatography would yield fewer identified chemicals than more sophisticated techniques such as gas chromatography-mass spectrometry (GC-MS), therefore, the number of chemicals per method were identified and listed, and (ii) the representation of species in the dataset. To highlight taxa and chemical groups which could be considered under-represented in the research, we listed all species of Carnivora and then determined whether they were present in the chemical signal literature as a first step. We then examined the proportion of species which had been investigated vs the total number of species in their Family, and the percentage of chemical signal literature representing each Family. This allowed us to identify potential shared features across taxa which have been investigated, e.g. whether they were known to use chemosignals, their geographic distribution, and their availability as subjects to researchers (captive population vs. wild only). While we are obviously limited to the data that has been published, this initial review of commonalities can usefully guide future work.

### Collation of data

Chemicals listed in collated publications were added to a bespoke database. These datasets were processed to remove misspellings and synonyms, and each chemical entity was appended with its CAS number (a unique code used for regulated chemicals given their potential hazard status), where possible. For each chemical, the molecular formula and chemical “type,” e.g. aliphatic hydrocarbons, carboxylic acids, esters, etc., of the substance were identified and listed in a separate table. To reduce errors, duplication, and redundancy, a list of misspelt and synonymous chemicals was built, and common errors were collated. Our database, which was built in Microsoft Access, allows chemicals to be associated with the species, reference publication, analysis method, the associated location on the animal, e.g. feces, urine, anal gland, etc., whether the animals sampled were wild or captive, and where known, the sex of the animal it was found in, and the function of the chemical where identified. This allows the database to be queried in different ways, for instance, to extract lists of chemical signals that have been found in female urine across a range of species.

### Collation of study characteristics

The sample size, collection methods, analysis methods, and reported chemical composition (where applicable) were extracted from each study. The basic metrics for each of these are presented in Table 1, and the full details of the publications identified for each species are given in Supplementary Table 1.

**Table 1.** **Characteristics identified in studies.** NB: Many publications covered multiple species.

Characteristic	*N*
Publications cited	112
Chemicals described	1,532 (by CAS number)
Species	66
Functions	14
Locations	14
Analytical methods	14
Associations of chemicals and species^a^	4,688

^a^Association is defined here as the presence of a chemical identified to a species, for example, indole was found in multiple species and products and was represented in 49 identified associations.

## Results

### Included studies

Data from 112 publications were collated, including 5 PhD theses and 107 peer-reviewed publications but no patents. The methodological details, lists of semiochemicals identified, and behavioral contexts investigated were entered into a bespoke database containing inter-linked tables. Some species were repeatedly sampled across papers while others were represented by a single publication.

### Differences in detailed data presented across studies

The number of chemicals reported within individual studies was affected by the analytical method employed and the availability of high-resolution analytical tools. However, there was also a significant effect of subjective choice concerning how many of the identified chemicals were presented: some authors chose to present only chemicals which they thought encoded information, whilst others presented all identified chemicals within the sample matrix regardless of whether they were believed to be chemical signals, or benign metabolites/byproducts. Academic theses tended to present more extensive lists of the chemicals than peer-reviewed publications, since they frequently included lengthy appendices that listed all chemicals found to be present in a given sample, whether or not they were biomarkers, differing between some identified group or sex. Thus, there was considerable variation in the available information for each species and we could not control for the variation that arose from differences in publishing conventions between peer-reviewed literature and student theses. Therefore, we could not form conclusions as to whether some species have more complex chemical signal interactions than others.

We identified six main sources of variation in the data presented across studies:

#### Variation based on analytical method.

Overall, there was a tendency to report more volatile organic compounds (VOCs) and semi-volatile organic compounds, and to a lesser extent compounds that can be made volatile by derivatization for detection by GC-MS. Careful consideration is required when selecting whether to utilize GC-MS or LCMS as the analytical method of choice as each method is more suitable for detecting different types of compounds. GC-MS is proficient for analyzing VOC such as small molecule metabolites or other compounds that can readily enter the gas phase (Theodoridis et al. 2011). nonvolatile organic compounds can be analyzed via GC-MS however this requires a derivatization step to enable these compounds to be volatilized (Theodoridis et al. 2011), this involves prior knowledge of the chemical identities of target analytes and as such is not appropriate for many chemical scent systems which remain uncharacterized. For nonvolatile organic compounds from small metabolites to proteins as analytes just need to be solubilized in a suitable carrier solvent. This is a simplified view of the myriad factors that influence analyte detection in GC and LCMS analysis, which is outside the scope of this review, however, it is worth highlighting that the method of chemical analysis will influence which analytes are detectable and may not necessarily be able to provide full coverage of all analytes involved in a complex scent system.

Many chemical analyses of chemical scents in mammals focus on volatile organic compounds, typically using GC-MS techniques (Soso et al. 2014). However, such approaches are unable to account for nonvolatile organic compounds that may act as important scent compounds. For example, it has been observed that mice utilize small communicative proteins called “major urinary proteins” which they secrete in much larger quantities in urine compared to other proteins (Roberts et al., 2014). As mice urine also contains a multitude of small metabolites also used for scent signals (Roberts et al. 2014), a pure GC-MS analysis will not reveal the full scope of chemical classes involved in these scent-based communication mechanisms. Thus, it is important for researchers to be aware of the suitability of an analytical method for detecting various chemical classes and also evaluate previous reports in a similar manner.

Relatively few studies reported analysis of nonvolatile organic compounds, e.g. amino acids, peptides, and proteins. These types of nonvolatile chemicals are best analyzed by solution-phase techniques, such as liquid chromatography-mass spectrometry (LC-MS) or nuclear magnetic resonance spectroscopy (Hoffman and Stroobant 2007). These techniques are increasingly prevalent in the metabolomics/proteomics fields and it seems inevitable that they will be applied much more extensively to the analysis of chemical signals in the near future, particularly since there are indications, in other species such as mice, that proteins are also involved in recognition of individuals despite being nonvolatile (Hurst et al. 2001; Nevison et al. 2003; Beynon and Hurst 2004). It has traditionally been assumed that only the most volatile compounds are used as chemical signals, but since it is now clear that nonvolatile compounds such as proteins may also be used (see Table 4) this should be reflected in the choice of analytical methods in future.

**Table 4.** Groups of chemicals and their associations with species and functions. Note that the numbers here are dependent on the analytical method used and therefore may not be representative. NB: number of reports does not equal number of publications (*N* = 112) as there were multiple species included in some publications which were counted as separate reports.

Classification	N of reports of presence in species	N of reports of associations with communicative function
Alcohols, phenols, and ethers	560	36
Aldehydes	441	28
Alicyclic hydrocarbons	88	2
Aliphatic hydrocarbons	336	19
Aromatic hydrocarbons	79	3
Carboxylic acids	953	73
Esters	417	34
Ketones	625	60
Nitrogenous compounds	679	60
Oxygenous heterocyclic compounds	199	9
Proteins and peptides	27	5
Sterols	54	37
Sulphurous compounds	386	36
Other ^a^	13	19

^a^The classification includes two halocarbons, a phosphate, a peroxide, and five organosilicon compounds.

#### Variation in available detail.

**Fig. 3.**
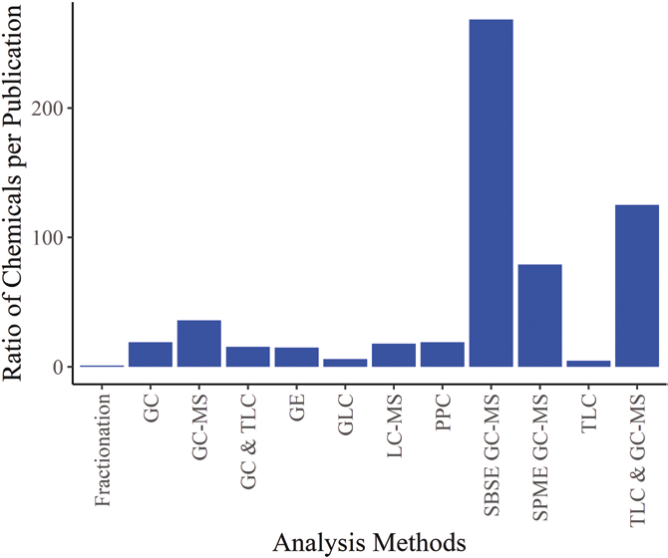
Ratio of identified/reported chemicals per publication for different analysis methods. Abbreviations: GC = gas chromatography, GLC = gas-liquid chromatography, GE = gel electrophoresis, LC = liquid chromatography, MS = mass spectrometry, PPC = paper partition chromatography, SBSE = stir bar sorptive extraction, SPME = solid-phase microextraction, and TLC = thin layer chromatography.

There were large variations in the number of chemicals found per study across the decades (a ratio of an average 1 chemical/publication in the 1890s through to an average of 85 chemicals/publication in the 2010s) which was likely due to improvements in the analysis method used (Fig. 3). Technological advances in mass spectrometry (MS) technology have proven to be especially important to the increase in the number of compounds and precision of identification with time. This is due to the unique characteristics of MS among analytical techniques, namely high molecular specificity (accurate molecular mass measurements), versatility in structural determination, high detection sensitivity, ease of up-stream coupling with chromatographic separation techniques, and the wide applicability to different sample types (polar, nonpolar, volatile, nonvolatile) (de Hoffman and Stroobant 2007).

#### Variation in species selection.

There was considerable variation in the selection of the species studied. Some species were studied repeatedly, with different analyses applied, and the results were presented in more than 5 publications, e.g. tigers (*Panthera tigris*). Other species were presented only in a single publication, and sometimes only in comparison to the focal species. This led to variations in the amounts of available data across taxa. Overall, the most poorly studied species were the Procyonoidae (raccoon) species, the Eupleridae (mongoose), and more surprisingly, the Phocidae (earless seals) and Otariidae (eared seals), which are pinnipeds (Table 2). Thus, it is difficult to assess conclusively whether any chemical signals are taxon specific or common across species when there is so much variation in which species, which chemical signals, and which functions have been studied.

**Table 2.** Species investigated, together with number of species per Family, and proportion of species for which some form of chemical signals analysis has been undertaken.

Family	Species in Family	Number of Species investigated	% Species investigated
Canidae (dogs, wolves, foxes)	35	8	22.9
Eupleridae (Malagasy mongooses)	10	1	10.0
Felidae (cats)	37	18	48.6
Herpestidae (mongooses)	37	5	13.5
Hyaenidae (hyenas)	4	3	75.0
Mephitidae (skunks, skunk-badgers)	11	3	27.3
Mustelidae (weasels, stoats)	54	11	20.4
Odobenidae (walrus)	1	0	0.0
Otariidae (eared seals)	14	2	14.3
Phocidae (earless seals)	19	1	5.3
Procyonidae (racoons)	18	1	5.6
Ursidae (bears)	9	2	22.2
Viverridae (civet, binturong, genets)	35	5	14.3

#### Variation in analytical detail reported.

There were differences across different studies in the level of specificity to which chemical compounds were identified. Identification varied from determining the chemical group, the general atomic formula, or specifying the isomeric forms present; some publications listed chemicals at only the broadest level, e.g. an “ester”; others listed a common name; while the most determinative defined the chemical formula and whether it was cis- or trans-isomer that was present using International Union of Pure and Applied Chemistry (IUPAC)-naming conventions. However, the use of IUPAC names was not universal, and this caused issues for initial comparison across papers (see below for a discussion of the importance of considering stereochemistry). Since many species have been investigated only in a single study, some of the chemical signals present are unlikely to have been detected because of either the chosen method of analysis, or the chosen biological medium studied, for example, feces, urine, etc., or methodological differences. These differences in the detection rates make conclusions somewhat tentative, particularly as most publications only listed chemicals that were looked for and found, thus chemicals not listed might have been present but not considered important or at subthreshold levels. Furthermore, if a chemical is difficult to extract and identify, or is present only in small quantities, its presence may be missed across multiple species. This makes it difficult to draw definitive conclusions about the presence, function, or use of chemical signals across taxa, since what was a reasonable degree of accuracy in the 1970s is generally too broad by today’s standards based on the cutting-edge molecular analysis technologies available.

Furthermore, studies investigating chemical signaling mechanisms can have several confounding challenges. These can range from attempts to describe responses that are complex and multi-variate, either behaviorally or biologically, to difficulties when analyzing the important signaling compounds which are only present within complex matrices (i.e. secreted or excreted substances) and/or present in low concentrations. These challenges are again further amplified in non-model study systems for which little to no literature exists, and therefore there are no data to use as a roadmap for future analyses. Although advances have been made in the biological and analytical chemistry disciplines, neither is capable of providing researchers with all the essential information required to produce accurate conclusions in isolation. Thus, there is an increasing need for strong collaborative efforts between biologists and chemists to match the chemical and functional data required to understand the exact nature of these signaling systems. A good example of the success of the collaborative approach comes from outside the carnivora; namely the characterization of sex pheromones released from the Australian orchids which rely on the sexual deception of male thynnine wasps (*Catocheilus* spp.) for pollination (Bohman et al., 2014, 2019). By coupling biological assaying with analytical chemistry methodologies, researchers were able to determine analyte characterization and functionality (e.g. Riffell et al., 2002). This could be considered a good model for other biologists and chemists looking to link chemicals and behavior in other species.

#### Variation in choice of sample source location.

**Fig. 4.**
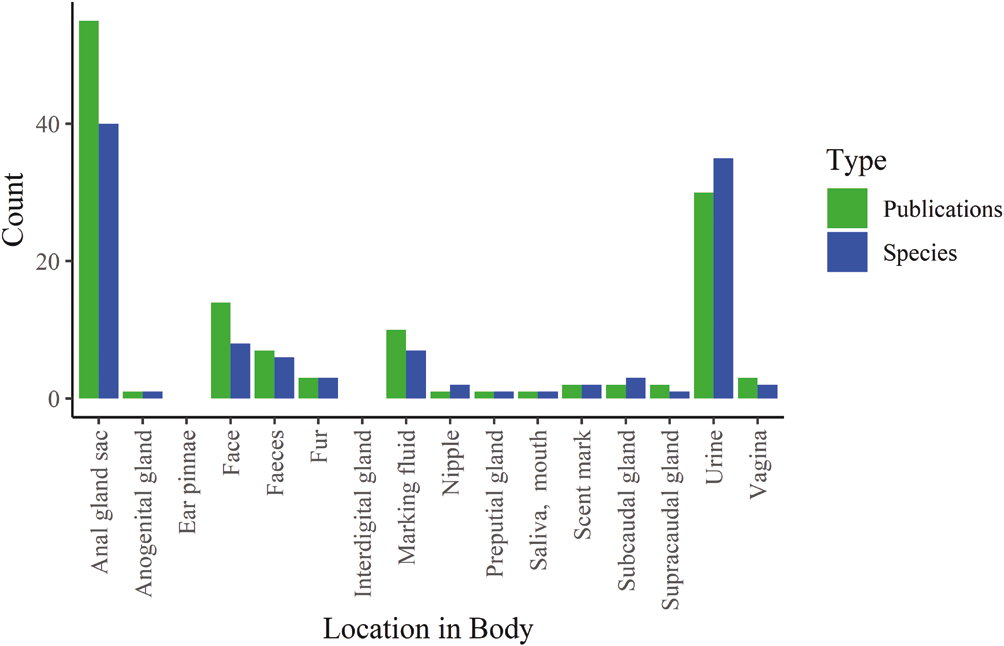
Number of locations of chemicals of interest and species recorded in the literature. Number of publications given in green and number of species investigated in blue for each location. Publications describing anal gland sac secretions were the most prevalent in the literature, however, only 40 of the 290 + species of Carnivora have had the contents of any anal gland sacs documented.

There was a preference for easily sampled matrices which are known to have overt odors (Fig. 4) or to be active in scent-behaviors, whilst the number of chemicals determined to be present varied widely by both type of analysis and sampling location (Table 3). Thus, anal gland sacs and pouches represent the foremost locations studied, followed by urine, marking fluid, and feces, whilst the chemicals present on fur and skin, in the preputial gland, facial glands, genitalia, subcaudal glands, supracaudal glands, pinnae, and interdigital glands were rarely or never analyzed in a given species (Fig. 4), despite an apparent breadth of chemical diversity identified in those studies that have analyzed them in other species (Table 3).

**Table 3.** Description of glands and secretions that have been investigated, with number of chemicals per sample, number of species which have been analyzed, and the calculated percentages of species analyzed and represented in literature.

Source	Chemicals found to be present	Species investigated	Total publications	Representation as % of all semiochemical publications
Anal gland sac	875	40	55	45.1
Anogenital gland	23	1	1	0.8
Ear pinnae	0	0	0	0.0
Face	119	7	4	3.3
Faeces	295	6	7	5.7
Fur	21	3	3	2.5
Interdigital gland	0	0	0	0.0
Marking fluid	198	7	10	8.2
Nipple	7	2	1	0.8
Preputial gland	47	1	1	0.8
Saliva / mouth	4	1	1	0.8
Scent mark	69	2	2	1.6
Subcaudal gland	19	3	2	1.6
Supracaudal gland	100	1	2	1.6
Urine	570	35	30	24.6
Vagina	13	2	3	2.5

#### Selective chemical reporting variation.

Many studies report the presence of either specific analytes or more broad chemical classes or formulae, without any demonstration of biological functionality. It is difficult to ascertain whether any of the chemicals identified play a significant role in the various signaling mechanism being investigated or if other, unidentified features were also important. Where functionality was identified, the studies rarely explored the stereochemistry of the signals. This is another important aspect that should be considered whilst reporting on chemical signaling compounds, since some biological mechanisms may rely on the detection of specific chemical isoforms for function. For example, sperm chemotaxis in the red abalone depends on the L isomer of tryptophan with the D isomer eliciting no response (Riffell et al. 2002). A final source of variation came from a general lack of information on which parts of the signal are active and how they link to the signal’s function, as few studies detailed the chemical identity of function-associated signals to this level.

### Collated results

We collated the data from the 112 sources. We then divided the literature and counted how many publications presented data on each species and which location. As can be seen in Table 3, many species still await investigation, and the focus has been on range marks, for example, products from the anal glands, urine, and marking fluid, over more intimate chemical signals.

We assigned each chemical signal within our overall dataset to specific groups depending on the chemical groups present within the molecule. These groups included, but were not limited to, alcohols, ethers, ketones, and proteins (see Table 4 for a summary). We then counted the number of chemicals per group and summarized the chemical groups represented and the number of associations between chemicals and species and their function in Table 4. We add the caveat that these numbers are dependent on the analytical method used, and as noted above, peptides or proteins, for instance, may be under-detected because the analytical techniques required to detect them were rarely used.

Furthermore, since chemical signals can be produced (i) from synthesis within specialized exocrine glands, such as the preputial gland (Apps et al. 2012) and the anal gland (Albone and Perry 1976; Theis et al. 2013), (ii) from environmental sequestration (Allen et al. 2017), and/or (iii) as the result of microbial symbiosis (Albone et al. 1977; Theis et al. 2012, 2013; Leclaire et al. 2017), there are difficulties in obtaining samples. In addition, chemical signals can be acquired from the environment by other means such as scent rubbing (Ryon et al. 1986; Clucas et al. 2008; Allen et al. 2017) and self-anointing (Charlton et al. 2020). The functional significance of related behaviors has been investigated in very few species and, in general, the process involved in the environmental acquisition of scents is not well understood or even documented for many species, with the assumption often made that scents are deposited rather than acquired from the environment. Nonetheless, we identified several distinct behavioral functions associated with the various sample matrices in these contexts, including specific active chemical signals within them. These include directed behavioral signals, e.g. appeasement, attraction, and territorial marking-defense, as well as demographic signals, including individual and group identity, sex, reproductive state, age, and even pair bond identity (see Supplementary Table 2).

### Commonalities across taxa

We explored the chemicals that were reportedly present in multiple species. Querying our database showed that 550 chemicals were found to be present in more than 1 species. Of these, 400 chemicals were found in more than one Family of Carnivora, and 39 were found in 5 or more Families (Table 5). The number of associated behavioral signal contexts for these ranged from 0 to 5, this evidently underestimates their behavioral relationships. Nine of the chemicals identified across 5 families have not been associated with any putative-identified functions, and this clearly reflects a gap in the research and not a lack of functionality. The putative identified functions are discussed in the next section.

**Table 5.** Chemicals which occur in more than 5 Families of Carnivora, total number of species they are present in, and number of associated behavioral signal functions proposed.

Chemical	N of Species	N of Families	N of Putative Identified Functions
Hexadecanoic acid	25	6	5
Benzaldehyde	24	7	5
Indole	28	6	4
Tetradecanoic acid	21	6	4
Dodecanoic acid	20	7	3
Propanoic acid	19	6	3
Oleic acid	13	5	3
2-Pentylfuran	10	5	3
Butanoic acid	23	7	2
Acetic acid	22	7	2
Octadecanoic acid	22	6	2
Nonanal	21	6	2
Pentanoic acid	21	6	2
2-Methylpropanoic acid	19	7	2
3-Methylbutanoic acid	19	7	2
Hexanoic acid	19	6	2
Nonanoic acid	18	6	2
2-Heptanone	16	5	2
Hexanal	16	5	2
Octanoic acid	15	6	2
Decanoic acid	14	5	2
2-Phenylethylamine	20	5	1
2-Methylbutanoic acid	16	7	1
Heptanoic acid	15	6	1
Pentadecanoic acid	15	6	1
Squalene	14	6	1
1-Hexadecanol	12	6	1
2-Undecanone	12	5	1
1-Hexanol	11	5	1
Limonene	7	5	1
Phenol	18	5	0
Decanal	17	6	0
Heptadecanoic acid	16	7	0
Cholesterol	12	5	0
Tridecanoic acid	12	5	0
1-Tetradecanol	10	5	0
4-Methylphenol	10	5	0
Hexadecane	7	5	0
Phenylacetaldehyde	5	5	0

### Chemical signals linked to specific behavioral functions

Having established which chemical signals are present and act as biomarkers across a range of species, it becomes possible to question which of these biomarkers are also bioactive signals and to question whether any of these bioactive signals have the same function in more than one species or family. Whilst many biomarker chemicals have been identified, only 252 have been identified as bioactive and relevant to specific behavioral contexts or functions. Amongst them, some were associated with multiple signaling regimens (Table 5): e.g. hexadecanoic acid is present in the territorial marks made by members of both the Felidae and Canidae, but it is also reported to be part of the appeasement signal of cats (Pageat and Gaultier 2003); it is also described in badgers as relevant to signal group identity (Buesching et al. 2016) and in lynx to signal individual identity (Vogt et al. 2016). It is not clear if it has multiple functions in the same species or if it is used for different behavioral contexts across species. It is likely, however, that this is a conserved evolutionary trait rather than a case of convergent evolution. As the number of reports of chemical analyses grows and the data are more easily collated, as in PUBCHEM (Kim et al. 2016) or CHEMSPIDER (Pence and Williams 2010) databases, it should become possible to examine the links between the use of these chemicals. Our current work highlights how wide a range of functions each can be involved in but is not able to establish whether these functions are repeated across taxa from the existing research.

Indeed, establishing the occurrence of common bioactive signals across taxa is particularly challenging because of the lack of detailed data on their presence and use in different species, even where there are data on their use in more than one. A single function was ascribed to 178 chemical signals (Table 5). However, since only 45 (40%) studies tested the association of specific chemicals with specific functions (e.g. exposing test subjects to a sample and recording behavioral responses), this probably represents a gross underestimate of the number of shared signals. By producing a consistent database of chemical signals, and their characterization as biomarkers and bioactive signals, we can enable greater awareness of this sort of sharing.

Typically, this begins with the identification of single chemicals as biomarkers. Although focusing on a singular molecule is limiting from a behavioral standpoint, it is important to remember that scent analysis is a combination of both biological and chemical analyses, and given the scent system in many taxa including those of Carnivora have little to no literature available, focusing on a singular compound provides an initial starting point in which to further develop and investigate non-model scent systems. Currently, there is a tendency to first investigate whether known chemical signals are present and potentially active before considering what else is present. This can be useful as it allows the direct comparison of the presence of biomarkers across taxa but is also problematic as it biases analysis towards these compounds: for example, since hexadecenoic acid has been identified in various studies, it is assayed specifically in many targeted/quantitative studies, whereas a potentially functional chemical signal from a group which has not been previously identified as functional may be missed or its presence overlooked due to no known link with a function. This can result in biased reporting within the literature, and one which is difficult to address, since it is easier to use existing knowledge as a roadmap to identifying likely chemical signals than it is to explore all potential chemical signals in untargeted, “discovery” workflows.

A list of products associated with functions is given in Table 6 (a full list of chemical signals with associated functions is given in Supplementary Table 2). Our review of the data that informs Table 6 demonstrates a further issue in so far as what has been identified in one species as an essential bioactive signal, e.g. the appeasement signal chemicals produced by both dog and cat nipples (Pageat and Gaultier 2003), may not have been investigated in a broader range of taxa. However, it can be seen that similar functions have been identified across a diverse range of media, e.g. bioactive signals of sex have been found in Egyptian mongoose anal gland secretions (Hefetz et al. 1984), domestic cat feces (Clapperton et al. 1988), and ferret urine (Zhang et al. 2005). Thus, it could be that sex is signaled in a range of different scents or that sex is signaled in all of these scents across species. However, there have not been enough studies of the bioactive products to establish whether this is the case.

**Table 6.** Bioactive signal functions of different chemical signals, their source location in the animal, and the relevant species evaluated.

Signal Type	Reported signal Function	Site of production in animal	Species	Citation
Behavioral	Appeasement	Nipple	Dog	(Pageat and Gaultier 2003)
			Domestic cat	(Pageat and Gaultier 2003)
	Neuter status	Urine	Maned wolf	(Jones, 2017)
	Stimulates overmarking	Urine	Red fox	(Apps and Weldon 2015)
	Territorial marking	Face	Domestic cat	(Pageat and Gaultier 2003)
		Marking fluid	Leopard	(Poddar-Sarkar 2004)
		Urine	African wild dog	(Jordan et al. 2015)
			Red fox	(Wilson et al. 1978)
Demographic	Age (juvenile status)	Anal gland sac	European otter	(Bradshaw et al. 2011 ; Kean, Müller, and Chadwick 2011)
		Faeces	Gray wolf	(Martín and Barja 2010)
	Group identity	Anal gland	European badger	(Buesching et al. 2016)
		Scent mark	Spotted hyena	(Hofer et al. 2011)
	Individual advertisement / recognition	Anal gland	Domestic cat	(Miyazaki et al. 2018)
			Ferret	(Clapperton, Minot, and Crump 1988)
		Urine	Eurasian lynx	(Vogt et al. 2016)
	Pair bond identity	Urine	African wild dog	Jordan et al. (2015)
	Reproductive status advertisement	Anal gland	Coyote	(Schultz et al. 2005)
			Gray wolf	Raymer et al. (2005)
		Urine	Dog	(Dzięcioł et al. 2018)
			Giant panda	(Wilson et al. 2020)
			Maned wolf	(Jones, 2017)
		Vagina	Dog	(Goodwin and Gooding 2006)
	Sex advertisement / recognition	Anal gland	Egyptian mongoose	(Hefetz, Ben‐Yaacov, and Yom‐Tov 1984)
			European badger	(Noonan et al., 2019)
			Ferret	(Clapperton, Minot, and Crump 1988)
			Indian mongoose	(Miyazaki et al. 2018)
			Meerkat	(Leclaire et al. 2017)
		Anogenital gland	Giant panda	(Zhang et al. 2008)
		Face	Domestic cat	(Pageat and Gaultier 2003)
		Faeces	Domestic cat	(Uetake et al. 2017)
		Fur / mane	Fossa	(Vogler et al. 2007)
		Urine	Ferret	(Zhang et al. 2005)
			Gray wolf	(Raymer et al. 1984)
	Species advertisement / encoding	Urine	Binturong	(Greene et al. 2016)
	Time of year / season	Anal gland	Giant panda	(Zhou et al. 2019)
		Fur / mane	Fossa	(Vogler et al. 2007)
		Urine	Gray wolf	(Raymer et al. 1984)

### Potential effects of diet and microbiota

The potential role of diet on microbiota and thus chemical signals is a relatively recent area of research interest. Since signaling associated with many species has only been studied in captivity, where diets are often quite different to those available to the species’ wild counterparts (Shukla et al. 2014), it is possible that samples from captive populations may not represent the true spectrum of chemical signals in the wild or may not contain key components which are derived from specific food sources. There is certainly a potential effect of diet on the perception of signals, for example, diet has been shown to influence the response of prey species to coyote (*Canis latrans*) urine, with meat-eating coyotes producing more aversive urine than vegetarian coyotes (Nolte et al. 1994). Due to the differences in data collection and analysis, it is not possible to draw firm conclusions from our study as to the importance of the presence or absence of certain chemicals following a dietary change or in situ versus ex situ sampling. However, it should be recognized that diet has the potential to influence both the signal and the response it elicits, for instance, mice recognize changes in diet for captive versus wild predators (Arnould et al. 1998). Studies of what triggers such responses are rare, but it is likely that it is a result of a complex interaction of different chemical signals rather than a single signal. Indeed, sheep respond with an aversion to dog or wolf feces but not to indole, a key signaling component of their feces, if it is presented in isolation (Arnould et al. 1998). The potential links between diet, behavioral responses, and chemical signals production and perception warrant much deeper investigation.

The “fermentation hypothesis” (Albone and Perry 1976) links the bacterial community present on and in an animal to the chemical signals that the animal produces, and provides direct evidence that chemical signal variation is affected by the animal’s diet, microbiome, and their interaction (Theis et al. 2012, 2013; Leclaire et al. 2017; Yamaguchi et al. 2019). The bacterial community appears to produce some of the chemicals through their own metabolic processes that are then used as chemical signals by the host animal, which cannot produce them independently (Theis et al. 2012, 2013; Leclaire et al. 2017; Yamaguchi et al. 2019). Since the role of microbiota in chemical signaling is not well understood (Ezenwa and Williams 2014), we suggest that this is an important area for future studies. The microbiota not only varies by bodily region within a species but can show consistent differences within a region based on sex, age, or group membership (Deusch et al. 2015; Pereira et al. 2020; DeCandia et al. 2021). Furthermore, these microbial communities can be affected by different factors, including diet, health, and reproductive status (Zoelzer et al. 2021). Microbiota communities are difficult to define as reliable features, since there is a large variation both within and between individuals, necessitating the need for large sample sizes for reliable information. However, studying the role, function, and mechanisms of interaction between diet, microbiota, and chemical signaling may provide an important step forward in our understanding of the complexity of animal communication.

Understanding the interaction between microbiota in different bodily regions and host secretions may be important, but first requires a baseline understanding of the compounds involved. Nonetheless, if microbial communities are key components of the production of chemical signals, and therefore animal communication, then there are perhaps important implications for animal welfare and management that are not widely appreciated. Antibiotics are a common tool in veterinary practice, but their role in altering the microbiota of the animal is not well appreciated (Menard et al. 2022). Nonetheless, it has been found experimentally that the application of antibiotics to female dogs in season, may affect their sexual attractiveness (Dziecioł et al. 2013). This is just a single example of an unexpected behavioral change that results from alterations to the animal’s microbiota, and it is likely that it is one of many. Further research is required to establish how antibiotics and probiotics may influence animal chemical signal production and response.

### Standardizing terminology: a proposed standard system for reporting chemical signal substances

Amongst the more than 1,500 chemicals identified, many were nitrogenous compounds (238); esters (207); alcohols, phenols, and ethers (194); ketones (179); and/or carboxylic acids (175). However, chemical identity was sometimes initially obscured by the use of synonyms for the same compound. Accordingly, we suggest the adoption of a standardized system for the reporting of potential chemical signals, as described here. This will help guide future research and aid comparisons between species.

For organic and inorganic chemical substances, our system presents three main identifiers: the IUPAC systematic name, the CAS number (if available), and the trivial/common name for the chemical (Table 7). Listing all three of these identifiers allows the reader to cross-reference the chemical substance and to recognize commonalities. Few, if any, authors present the IUPAC systematic name for well-known substances such as squalene or menthol, yet it is important to not rely on trivial/common names. The three identifiers should be reported within a body of work (e.g. journal article, book) or in additional/supplementary materials to the body of work (e.g. addendum, supplementary information sheet).

**Table 7.** Examples of the Reporting System.

Trivial name	IUPAC systematic name	CAS number	Molecular formula	Molecular mass (g/mol)^a^
Inorganic substances				
Ammonia	azane	7664-41-7	NH3	17.03
Octasulfur	octathiocane	10544-50-0	S8	256.5
Organic substances				
Benzene	benzene	71-43-2	C6H6	78.11
Indole	1H-indole	120-72-9	C8H7N	117.15
Squalene	(6E,10E,14E,18E)-2,6,10,15,19,23-hexamethyltetracosa-2,6,10,14,18,22-hexaene	111-02-4	C30H50	410.7
Menthol	5-methyl-2-propan-2-ylcyclohexan-1-ol	1490-04-6	C10H20O	156.26
Proline	(2S)-pyrrolidine-2-carboxylic acid	147-85-3	C5H9NO2	115.13
Uroguanylin	H-Gln-Glu-Asp-Cys-Glu-Leu-Cys-Ile-Asn-Val-Ala-Cys-Thr-Gly-Cys-OH^b^	N/A^c^	C61H101N17O25S4	1600.8

^a^The unit of the molecular weight should be stated.

^b^For peptide nomenclature, the IUPAC systematic name is proposed for peptides consisting of five or fewer residues, the IUPAC three-letter symbol system for peptides consisting of between six and twenty residues, and the IUPAC one-letter symbol system for peptides consisting of more than twenty residues.

^c^Many substances have not been assigned a CAS number.

Additionally, in studies that are proposing chemical characterizations, further data should be supplied so that reviewers and future readers are able clearly to assess the validity of any chemical characterization put forth. The supplementary material that should be supplied is wholly dependent on the chemical analysis performed to characterize the candidate analyte in question. In targeted LC-MS or GC-MS workflows this may be as straightforward as supplying the retention time and MS data of a corresponding authentic chemical standard. However, these supplementary chemical data are most important for untargeted LC-MS or GC-MS workflows in which mass spectral databases are used to provide tentative characterizations for detected analytes. The algorithms used to search these databases typically use some form of identification score to suggest confidence between the obtained mass spectra and those within the database. However, these approaches are prone to producing false discoveries and if taken at face value by less experienced practitioners these results can be misleading and potentially go unnoticed by reviewers (Theodoridis et al. 2023). Thus, researchers must carefully evaluate chemical characterization obtained through these types of workflows. A goal of this review is to highlight the need for a standardized method in which to report chemical names that are easy to grasp for readers from varying disciplines. Discussing concerns with chemical identification is, however, beyond the scope of this review. Nonetheless, Theodoridis et al. (2023) have published a comprehensive review on this topic in metabolomic studies and it clearly explains the pitfalls and methods to address these in future studies.

The latest recommendations from the IUPAC on terminology and nomenclature of organic and inorganic substances are available at www.iupac.org and access to the latest CAS number information from the Chemical Abstracts Service (CAS), a division of the American Chemical Society (ACS), is available at www.cas.org and www.commonchemistry.org.

## General discussion

Identification of chemicals by their IUPAC name revealed 550 chemicals common to two or more Carnivoran species and 169 known to occur in more than five species. Most published work focused on identifying the presence or quantity of chemicals rather than associating them with behavioral functions, and so it cannot be assumed that all biomarkers are bioactive signals, only that they have the potential to be so. Only by documenting all constituents of the matrices investigated may we identify key factors that might be bioactive.

Nonetheless, 178 chemicals were identified as bioactive signals, and of these, 30 were associated with more than one function. Hexadecanoic acid, indole, and benzaldehyde were found to encode information relevant to multiple behavioral contexts and were also found in the largest number of investigated species. Whilst signals encoded at a binary level (i.e. through their presence/absence) are the easiest to demonstrate; quantitative signals relating to individuality, sex, or dominance status have also been frequently identified. Carboxylic acids, ketones, and nitrogenous compounds were most often identified with functions, but this may reflect preference in the analytical methods used, as described above.

Chemical signaling can be present in numerous biological sample matrices (urine, glandular secretions, etc.) depending on the specific signaling mechanisms. The most frequently studied sample matrices are derived from those well known for their role in scent signaling, e.g. urine and anal gland products, whilst there was comparatively little research into other sample matrices collected from glandular structures, such as the subcaudal gland and interdigital glands, despite their potential to harbor important signals. The studies of anal gland products and urine highlight the broad range of chemicals present which may be used as signals in a large number of behavioral contexts, from aggression to affiliation, and indicating a wide range of demographic statuses from the very broad, e.g. species, to the current physiological age and emotional state of the individual.

Many species had the same chemicals (e.g. indole, hexadecanoic acid, and benzaldehyde), present in equivalent products (e.g. anal gland secretions, urine), suggesting an ancient phylogeny to chemical communication, with similar chemicals encoding similar information across related species, for example, sex, age, and reproductive status (see Supplementary Table 2). Likewise, decanoic acid forms part of a signal for group identity in both spotted hyenas and European badgers. There may also be common elements to functionally important chemical signals, such as the appeasement signals used across species: these are often a combination of octadecanoic acid, oleic acid, and hexadecanoic acid; however, there may also be species-specific elements which enhance this as suggested by Prior and Mills (2020). In a rare case of using semiochemicals to determine phylogeny, Bininda-Emonds et al. (2001) examined the chemical signals of *Felidae* and built a phylogeny based on the traits which were relatively consistent with other phylogenies. They found around 70% of the 400 identified “matrix characters” of lipid compounds which were phylogenetically informative. However, they did not investigate whether the use of the shared chemical signals was functionally similar or list which these were beyond broad categories of “lipid, glycolipid” etc.

Here, we have limited our review to Carnivora, but model organisms such as mice have extensive documentation of their chemical signals, including bioactive signals. Some of these are available in databases, such as PUBCHEM (Kim et al. 2016) and CHEMSPIDER (Pence and Williams 2010). Chemical signals may also have important effects between species where there is biological relevance: for example, mice respond fearfully to the pyrazine scent present in both cat and wolf urine (Berton et al. 1998; Osada et al. 2013, 2015).

### Limitations within the dataset

The variation in data reporting that we identified in the literature were due primarily to limitations in the available analytical techniques to researchers, with early publications less able to identify certain chemical components of sample matrices. Thus, research relying on early analytical methods like fractionation (Aldrich, 1896; Aldrich and Jones, 1897) identified fewer chemicals than later studies which could take advantage of GC-MS, even when researching the same genus or species (Wood, 1990; Wood et al., 2002).

A further source of potential variation in reporting comes from the choice of species and substances to investigate. The subjective perception of the likely importance of scent communication in the native habitats of species appeared to play a role in the selection of many species, i.e., there was a preference towards the obvious species such as skunks and foxes, while few publications investigated the chemical signals of marine mammals, despite intense interest in their overall behavior. This could be attributed in part to a perception that any species living in water might be unlikely to communicate via chemicals and thus it is not worth investigating them further. However, it should be noted that chemical communication is in fact extremely widespread within aquatic environments (Bronmark and Hansson 2000), with fish, for example, routinely making use of chemical signals during social interactions including mate selection (Yambe et al. 2006) and shoaling (Mann et al. 2003). A recent paper on bottlenose dolphins (*Tursiops truncatus*) also found that they cross-modally associate the signature whistle and urine plume of individuals (Bruck et al. 2022). Moreover, many marine carnivoran species haul out of the water to breed on land, thus they are not always communicating in an aquatic environment, and there is potential for chemical communication on land through the air (Wierucka et al. 2019). Tests of mother-infant recognition in Australian sea lion pups (*Neophoca cinerea*) have shown that it is partly based on scent recognition (Wierucka et al. 2019). Species distribution and geography were also influential on which animals were researched as species in Madagascar, i.e. the Eupleridae, were similarly under-represented. Other explorations of scent signals have demonstrated marked differences between wild and captive populations in such diverse taxa as tamarins (*Saguinus i. subgrisescens*) (Poirier et al. 2023), owl monkeys (*Aotus spp.*) (Spence-Aizenberg et al. 2018), and mice (Robertson et al. 1997).

One of the challenges we faced when undertaking this review was the diversity of terminology used for the chemicals and for their functions. The same chemical could have up to 10 synonyms (e.g. n-octadecanoic acid), and could be listed as merely a chemical component, pheromone, or given one of a number of other terms, potentially leading to confusion over its presence in (i) other species and (ii) use by different species. This was solved here, by listing all chemicals in the database by their IUPAC name and collapsing the different names into a single listing. This diversity has proven to be problematic before. As mentioned above, Osada and colleagues (Osada et al. 2013, 2015) identified that mice displayed fear behavior in response to the pyrazine analogues in wolf urine and ascribed this to a specific fear of wolves. However, it is likely that the mice were responding to pyrazine as a common component of predator scent since it is also present in other Carnivoran urine, including that of lynx (*Lynx rufus*) (Mattina et al. 1991) and male Bengal cats (*Felis catus × Prionailurus bengalensis*) (Yamaguchi et al. 2019). It is therefore important to be mindful of ascribing specific functions on the basis of limited evidence. The promotion of the data and nomenclature standards used to develop our database should help to address the issues described here, with the ability to search for synonymous chemicals. We have discussed one potential system for addressing this in the section above “A Proposed Standard System for Reporting Chemical Signals Substances.”

### Extraction and analytical methods

The choice of extraction and chemical detection methods for chemical analysis of biological matrices is paramount to correctly identify important analytes of interest relating to the biological question at the heart of the project. All these techniques can be considered as tools within a chemist’s toolbox and thus each will come with their own uses and limitations. Hence, careful consideration of which techniques will be implemented in these forms of chemical analyses is of the upmost importance prior to any form of experimental or preparatory work being conducted, as this choice will have a significant effect on what analytes can ultimately be detected and the sensitivity and specificity of the detection. From the literature of the chemical analyses performed on various scent systems, a wide variety of collection protocol (solid samples (e.g. feces, hair, etc.), liquid samples (e.g. blood, urine, etc.) and gas samples (e.g. breath and air sampling, etc.), extraction methods (e.g. liquid–liquid extraction, solid-phase extraction, etc.) and chemical analysis methods (i.e. gas or liquid chromatography tandem mass spectrometry in various forms; see Supplementary Table 1) were used. Ultimately, the choice of sample collection, preparation, and chemical analysis technique needs to be decided based on the requirements of the scent/biological system being studied, but it seems that it is often based on immediate/local availability of analytical infrastructure. In targeted chemical analyses, methods can be tailored to target specific analytes or analytes of a specific chemical class, which are known or thought to be significant (e.g. Hendriks et al. 1995). However, due to the difficulties of quantitatively assessing analytes beyond presence/absence, many studies focus simply on a qualitative approach in which to demonstrate the presence of an important analyte. As qualitative chemical analyses are comparatively simpler to perform than quantitative chemical analyses, they are useful as an initial step to first confirm the presence of important analytes (Aldrich 1896; Andersen and Bernstein 1975) which can be refined (Wood 1990) or reconsidered (Wood 1999). The use of a quantitative chemical analysis requires first robust collection and sample preparation methods prior to chemical analysis. However, targeted approaches rely on some amount of biological and chemical information being known prior to project commencement (Wood 1990; Jones 2017).

### Combining chemical and biological analyses

Determining suitable chemical analyses is particularly challenging in systems where there is strong evidence that a biological system is being chemically mediated, but there is no prior chemical information available to guide the choice. Thus, an untargeted chemical analysis approach may be required. These approaches come with significant challenges that must be carefully considered (Schrimpe-Rutledge et al. 2016; Cui et al. 2018). In recent times, “Omic” based workflows, utilizing high-resolution mass spectrometers, such as those based on orbitrap or time-of-flight (ToF) analyzers, have provided researchers with powerful tools with which to conduct untargeted chemical analysis to begin identifying biologically significant analytes (Schrimpe-Rutledge et al. 2016; Winder et al. 2022). However, despite advancements in MS and data analysis methods, MS is incapable of providing any information on the biological significance of any detected analytes. Thus, researchers exploring more complex chemical signaling mechanisms should look to design studies in which to leverage and integrate the strengths of analytical chemistry and behavioral/biological analyses to achieve a holistic perspective on how these chemical systems operate. Furthermore, in systems in which strong evidence supports that a biological response is mediated through chemical signaling, but for which little or no chemical information exists in the literature, such as in non-model taxa systems, collaborative efforts may be the only way to identify the relevant analytes mediating these systems. Thus, biological and chemical analysis methods should be designed to work in tandem, with a joint focus on the analytes of significance and how they can be connected to the biological response being investigated (as illustrated by Bohman et al. 2014, 2019).

### Future directions

More than 45% of the studies covered explored anal gland sacs and pouches and their contents, whilst few or no publications explored the presence of potential chemical signals in pinnae, interdigital glands, faces, fur/pelages, mouths and saliva, subcaudal glands, supracaudal glands, or the air surrounding the animal. Thus, many potential chemical signals are yet to be identified let alone their function explored. Indeed, there is much still to be discovered about animals’ use of overtly communicative gestures such as rubbing, scent-marking, and scratching. There are likely many chemicals that are linked to these actions, but it is not clear how important they are to the signals or if they modify the message sent.

Amongst carnivora, Bininda-Emonds et al. (2001) demonstrated how using chemical signals can inform phylogenies of the Felidae, showing that evolutionary history traces differences in the presence of lipids. However, this is an isolated case, although extensive work has been done in other taxa to determine shared chemical signals, at least as biomarkers, and how they have been affected by the species’ evolutionary history. Here, we suggest that as there are 39 chemicals (Table 5) shared across five or more families within the Carnivora it is likely that this chemical-based phylogenetic approach would be informative across the taxa in relation to both the presence and use of chemical signals. However, the role of microbiota in producing these commonalities should also be considered (Ezenwa and Williams 2014; Maraci et al. 2024).

Sex-specific chemical signals and related behaviors have been identified in some species, but again this is an under-explored area. Many publications do not state, or do not investigate, whether the chemicals are present in both sexes or only one, or to what extent quantities may vary within and between the sexes. However, sex-specific chemical signals have been identified in a number of species (Supplementary Table 2). It is clear that chemical signals may perform complex functions in sexual competition and reproductive choices, with animals encoding dominance or reproductive status in chemical signals (Stowers and Logan 2010; Greene et al. 2016; Dzięcioł et al. 2018), which result in clear physiological and behavioral responses in others. This is particularly evident in reproductive signaling, since the mechanisms that induce estrus, initiate mating, or inform mate choice, are often poorly understood. However, the central neurological processes underpinning this are even less well understood, although an important role via affective systems in the brain biasing behavior has been postulated to underpin their clinical potential (Mills et al. 2013). This perspective is different from the traditional focus on these chemicals as trigger stimuli of behavior (or fixed action patterns which underpinned early views of pheromones in insects and sexual behavior in other species (Karlson and Lüscher 1959)).

Another under-investigated aspect of chemical signaling is the amount of time that the signals are active (fade out time), the maximum signal rise time, and how these may affect response behaviors. While the importance of fade out time has been established in a small number of carnivore species, e.g. Ethiopian wolves (*Canis simensis*) (Sillero-Zubiri and Macdonald 1998) and gray wolves (*Canis lupus*) (Wolfram 2013a), it remains unexplored in most other species. However, this is a key aspect of animal communication and one that may be important to the role of different analytes within the signal. This review has not covered bioinformatic frameworks, but this is a notable area that should be explored in the future.

The relationship between bacteria and chemical signal production is also poorly documented in most species and its potential link to behavior in nonhuman animals is even more poorly understood, but undoubtedly of enormous scientific and potentially public health interest. We suggest that a combinatorial approach of multifaceted chemical analysis coupled with microbiota identification and detailed behavioral studies would be a useful approach to linking the bacteria with chemical signals and their functions.

## Conclusion

Potential chemical signals have been found in every species of Carnivora that has been investigated. The complexity of chemical communication, the key components, and even the behavioral contexts for it, are poorly documented for many species and entirely lacking for some Families. However, the studies which have been conducted demonstrate that a large amount of information is encoded in chemical signals and there exist extensive commonalities across taxa. This should be a focus of future work. Carnivora were shown to use chemical signals in a broad range of communication contexts and to produce a large number of potential signals both through their presence and quantity. A broad range of chemicals were identified to be (i) present and (ii) function as chemical signals across 66 species of Carnivora. Several shared the same behavioral function in many species, suggesting a possible shared phylogeny of chemical signal communication in mammals, though further work is required to establish this conclusively.

Overall, deposited products, particularly anal sac gland products, urine, feces, and marking fluid, were more likely to be analyzed in the literature, with comparatively few studies focusing on other areas where the chemical signals remain on the body instead of the habitat, such as the face, ears, tail glands, or saliva, despite their potential importance to communication. Similarly, chemical analyses were often aimed at the identification of volatile compounds using GC-MS, thus the significance of peptides, proteins and other nonvolatile compounds may be underestimated.

Proteomic, metabolomic and lipidomic analysis at present are not easily mapped back to the genome and this is a shortcoming when considering the entirety of how a biological system functions. However, it should be noted that the genome is relatively static compared to the proteome, with certain proteins only being expressed under specific conditions making mapping the proteome and relating it back to the genome a complex endeavor (Ahrens et al. 2010). Thus, although at present mapping proteins, lipids and metabolites back to the genome is difficult, omic base chemical analyses are still powerful tools that enable researchers to begin identifying and characterizing candidate analytes from complex biological or environmental samples.

Future research should focus on utilizing the full potential provided by coupling state-of-the-art biological, genetic, genomic, metagenomic, and analytical chemistry methodologies to provide a greater appreciation of how chemical signaling mechanisms mediate various biological and behavioral responses across taxa. Modern omic studies and bioinformatic analyses alongside the rising potential of AI provide enormous potential for understanding complex biological systems, with a study from being able to demonstrate metabolomic differences in neurotransmitters, glucose metabolism, polyamides, and phospholipids in the skeletal muscles of young and aged mice (Uchitomi et al. 2019). Advances in bioinformatics, software packages, and potentially AI, for analyzing complex MS-based omic data (Chen et al. 2020, 2022; Lam et al. 2021) provide great promise for future chemical scent analysis.

There is evidence, e.g. in mice (Hurst and Beynon 2004), that quantity (absolute as well as relative in relation to other chemicals) as well as physical presence can all affect behavioral response, and it seems reasonable to suggest this may apply to carnivora as well (Hurst and Beynon 2004). Indeed, in red foxes (McLean et al. 2021), there is some evidence that the quantity and proportion of the analytes in the medium may convey information. This suggests that more consideration should be given to the proportion and quantity of analytes when considering their potential role as bioactive signals. There is also a need for additional work on the potential significance of quantitative levels of chemicals, and their proportions in complex matrices, rather than considering chemical signals as uni-chemical, binary or ungraded messages. We believe that once analytes are identified, it is important to validate their role as signals by assessing their role in mediating animals’ behavior and whether presence equals signal function. This can only be truly done in whole organismal systems.

Finally, the wealth of research demonstrates that chemical signals are a key communication tool both within and between species and that much information can be gained from their investigation. We suggest that the order Carnivora may share a repertoire of chemical signals, including scents, and that different taxa may use the same chemical signals for similar behavioral contexts. Thus, by establishing the behavioral function in one species, it can be posited that a similar function will be found in others. The documented complexity of chemical signals and the number of behavioral contexts in which they are used suggest that they are critically important to communication in Carnivora and deserve in-depth attention in future. In sum, it is fair to suggest that we tend to grossly underestimate the influence of chemical signals on carnivore behavior.

## Supplementary Material

bjaf019_suppl_Supplementary_Table_S1

bjaf019_suppl_Supplementary_Table_S2

## Data Availability

The data underlying this article are available in the article and in online supplement at the OSF site.
