# Supplementary material for: Common scents? A review of potentially shared chemical signals in the order Carnivora
Source: Chem Senses. 2025 Jun 9;50:bjaf019. doi: 10.1093/chemse/bjaf019 (PMC12228039; doi:10.1093/chemse/bjaf019)
Supplement: bjaf019_suppl_Supplementary_Table_S1 [file bjaf019_suppl_supplementary_table_s1.docx]

**ESM**

**Table 1** **Summary of literature reviewed by species, with product, analysis method, situ status, and citing publication listed.**

| Species common name | Species Latin name | Location in animal | Captive or Wild | Analysis method | Citing publication |
| --- | --- | --- | --- | --- | --- |
| Aardwolf | *Proteles cristata* | Anal gland sac / pouch | Wild | GLC-MS | (Apps et al., 1989a) |
| African lion | *Panthera leo* | Anal gland sac / pouch | Captive | TLC GC-MS | (Albone and Gronneberg, 1977) |
| African lion | *Panthera leo* | Anal gland sac / pouch | Captive | TLC GC-MS | (Albone and Perry, 1976) |
| African lion | *Panthera leo* | Face | Captive | SBSE | (Soini et al., 2012) |
| African lion | *Panthera leo* | Fur / mane | Captive | GC - TLC | (Poddar-Sarkar et al., 2007) |
| African lion | *Panthera leo* | Marking fluid | Captive | SPME GC-MS | (Soso and Koziel, 2017) |
| African lion | *Panthera leo* | Urine | Captive | GC-MS | (Andersen, and Vulpius, 1999) |
| African lion | *Panthera leo* | Urine | Captive | GC-MS | (Apps et al., 2012) |
| African lion | *Panthera leo* | Urine | Captive | LC-MS | (Ferrero et al., 2011) |
| African lion | *Panthera leo* | Urine | Captive | PPC | (Datta and Harris, 1953) |
| African wild dog | *Lycaon pictus* | Anal gland sac / pouch | Wild | GC-MS | (Apps et al., 2012) |
| African wild dog | *Lycaon pictus* | Faeces | Wild | GC-MS | (Apps et al., 2012) |
| African wild dog | *Lycaon pictus* | Preputial gland and tuft | Wild | GC-MS | (Apps et al., 2012) |
| African wild dog | *Lycaon pictus* | Urine | Captive | GC-MS | (Apps et al., 2012) |
| African wild dog | *Lycaon pictus* | Urine | Wild | GC-MS | (Jordan et al., 2016) |
| American mink | *Mustela vison* | Anal gland sac / pouch | Captive | GC | (Schildknecht et al., 1976) |
| American mink | *Mustela vison* | Anal gland sac / pouch | Captive | TLC GC-MS | (Bernstein, 1979) |
| American mink | *Mustela vison* | Anal gland sac / pouch | Captive | TLC GC-MS | (Sokolov et al., 1980) |
| American mink | *Mustela vison* | Anal gland sac / pouch | Wild | GC-MS | (Brinck et al., 1983) |
| Antarctic fur seal | *Arctocephalus gazella* | Face | Wild | GC-MS | (Stoffel et al., 2015) |
| Asiatic lion | *Panthera leo persica* | Marking fluid | Captive | TLC | (Brahmachary and Singh, 2000) |
| Asiatic lion | *Panthera leo persica* | Urine | Captive | GE-MS | (Mclean et al., 2007) |
| Australian sea lion | *Neophoca cinerea* | Fur / mane | Wild | GC-MS | (Wierucka et al., 2019) |
| Australian sea lion | *Neophoca cinerea* | saliva / mouth | Wild | GC-MS | (Wierucka et al., 2019) |
| Banded mongoose | *Mungos mungo* | Anal gland sac / pouch | Wild | GC-MS | (Jordan et al., 2010) |
| Bengal cat | *Prionailurus bengalensis* | Anal gland sac / pouch | Captive | SPME GC-MS | (Yamaguchi et al., 2019) |
| Binturong | *Arctictis binturong* | Anal gland sac / pouch | Captive | GC-MS | (Weldon et al., 2000) |
| Binturong | *Arctictis binturong* | Urine | Captive | GC-MS | (Greene et al., 2016) |
| Black-backed jackal | *Canis mesomelas* | Anal gland sac / pouch | Wild | GC-MS | (Apps et al., 2012) |
| Bobcat | *Lynx rufus* | Urine | Captive | GC-MS | (Mattina et al., 1991) |
| Bobcat | *Lynx rufus* | Urine | Captive | LC-MS | (Ferrero et al., 2011) |
| Bobcat | *Lynx rufus* | Urine | Wild | PPC | (Hendriks et al., 1995) |
| Brown bear | *Ursus arctus* | Anal gland sac / pouch | Wild | GC-MS | (Rosell et al., 2011) |
| Brown bear | *Ursus arctus* | Urine | Not stated | LC-MS | (Ferrero et al., 2011) |
| Cape genet | *Genetta tigrina erlangeri* | Anal gland sac / pouch | Captive | TLC GC-MS | (Jacob and Schliemann, 1986) |
| Cape genet | *Genetta tigrina erlangeri* | Urine | Captive | PPC | (Datta and Harris, 1953) |
| Caracal | *Caracal caracal* | Urine | Wild | GC-MS | (Goitom, 2017) |
| Cheetah | *Acinonyx jubatus* | Marking fluid | Captive | TLC | (Brahmachary and Singh, 2000) |
| Cheetah | *Acinonyx jubatus* | Marking fluid | Captive | TLC | (Poddar-Sarkar and Brahmachary, 1997) |
| Cheetah | *Acinonyx jubatus* | Urine | Both | GC-MS | (Burger et al., 2006) |
| Cheetah | *Acinonyx jubatus* | Urine | Captive | LC-MS | (Ferrero et al., 2011) |
| Clouded leopard | *Neophilis nebulosa* | Urine | Captive | GE-MS | (Mclean et al., 2007) |
| Coati | *Nasua* | Urine | Captive | LC-MS | (Ferrero et al., 2011) |
| Common genet | *Genetta genetta* | Anal gland sac / pouch | Captive | TLC GC-MS | (Jacob and Schliemann, 1986) |
| Coyote | *Canis latrans* | Anal gland sac / pouch | Captive | GC-MS | (Preti et al., 1976) |
| Coyote | *Canis latrans* | Anal gland sac / pouch | Wild | GC-MS | (Schultz et al., 1988) |
| Coyote | *Canis latrans* | Urine | Both | LC-MS | (Ferrero et al., 2011) |
| Dog | *Canis familiaris* | Anal gland sac / pouch | Captive | GC-MS | (Apps et al., 2012) |
| Dog | *Canis familiaris* | Anal gland sac / pouch | Captive | GC-MS | (Natynczuk et al., 1989) |
| Dog | *Canis familiaris* | Anal gland sac / pouch | Captive | GC-MS | (Preti et al., 1976) |
| Dog | *Canis familiaris* | Faeces | Captive | GC-MS | (Arnould et al., 1998) |
| Dog | *Canis familiaris* | Nipple | Captive | Not given | (Pageat and Gaultier, 2003) |
| Dog | *Canis familiaris* | Urine | Captive | GC-MS | (Apps et al., 2012) |
| Dog | *Canis familiaris* | Urine | Captive | GC-MS | (Dzięcioł et al., 2018) |
| Dog | *Canis familiaris* | Urine | Captive | GC-MS | (Schultz et al., 1985) |
| Dog | *Canis familiaris* | Urine | Captive | PPC | (Datta and Harris, 1953) |
| Dog | *Canis familiaris* | Urine | Captive | SBSE | (Wolfram, 2013) |
| Dog | *Canis familiaris* | Vagina | Captive | GC-MS | (Dzięcioł et al., 2018) |
| Dog | *Canis familiaris* | Vagina | Captive | GC-MS | (Goodwin et al., 1979) |
| Domestic cat | *Felis domesticus* | Anal gland sac / pouch | Captive | GC-MS | (Preti et al., 1976) |
| Domestic cat | *Felis domesticus* | Anal gland sac / pouch | Captive | GC-MS | (Miyazaki et al., 2018b) |
| Domestic cat | *Felis domesticus* | Anal gland sac / pouch | Captive | GC-MS | (Miyazaki et al., 2018c) |
| Domestic cat | *Felis domesticus* | Face | Captive | Not given | (Pageat and Gaultier, 2003) |
| Domestic cat | *Felis domesticus* | Faeces | Captive | GC-MS | (Uetake et al., 2018) |
| Domestic cat | *Felis domesticus* | Nipple | Captive | Not given | (Pageat and Gaultier, 2003) |
| Domestic cat | *Felis domesticus* | Urine | Captive | Not given | (Westall, 1953) |
| Domestic cat | *Felis domesticus* | Urine | Captive | GC-MS | (Apps et al., 2014) |
| Domestic cat | *Felis domesticus* | Urine | Captive | GC-MS | (Miyazaki et al., 2006) |
| Domestic cat | *Felis domesticus* | Urine | Captive | GE-MS | (Mclean et al., 2007) |
| Domestic cat | *Felis domesticus* | Urine | Captive | LC-MS | (Ferrero et al., 2011) |
| Domestic cat | *Felis domesticus* | Urine | Captive | PPC | (Datta and Harris, 1953) |
| Domestic cat | *Felis domesticus* | Urine | Captive | PPC | (Hendriks et al., 1995) |
| Egyptian mongoose | *Herpestes ichneumon* | Anal gland sac / pouch | Captive | GC-MS | (Hefetz et al., 1984) |
| European badger | *Meles meles* | Anal gland sac / pouch | Both | GC | (Davies et al., 1988) |
| European badger | *Meles meles* | Anal gland sac / pouch | Wild | GC-MS | (Buesching et al., 2016) |
| European badger | *Meles meles* | Anal gland sac / pouch | Wild | GC-MS | (Noonan et al., 2019) |
| European badger | *Meles meles* | Subcaudal gland | Wild | GC-MS | (Buesching et al., 2002) |
| European otter | *Lutra lutra* | Anal gland sac / pouch | Captive | SPME GC-MS | (Kean et al., 2015) |
| European otter | *Lutra lutra* | Anal gland sac / pouch | Wild | GC-MS | (Bradshaw et al., 2001) |
| European otter | *Lutra lutra* | Anal gland sac / pouch | Wild | SPME GC-MS | (Kean, 2012) |
| European otter | *Lutra lutra* | Anal gland sac / pouch | Wild | SPME GC-MS | (Kean et al., 2011a) |
| European otter | *Lutra lutra* | Faeces | Wild | SPME GC-MS | (Kean, 2012) |
| Ferret (domestic) | *Mustela putorius furo* | Anal gland sac / pouch | Both | GC-MS | (Crump, 1980a) |
| Ferret (domestic) | *Mustela putorius furo* | Anal gland sac / pouch | Captive | GC | (Clapperton et al., 1988) |
| Ferret (domestic) | *Mustela putorius furo* | Anal gland sac / pouch | Captive | GC-MS | (Crump and Moors, 1985) |
| Ferret (domestic) | *Mustela putorius furo* | Anal gland sac / pouch | Captive | SBSE | (Zhang et al., 2005) |
| Ferret (domestic) | *Mustela putorius furo* | Anal gland sac / pouch | Wild | GC-MS | (Brinck et al., 1983) |
| Ferret (domestic) | *Mustela putorius furo* | Urine | Captive | SBSE | (Zhang *et al.*, 2005) |
| Ferret (domestic) | *Mustela putorius furo* | Urine | Not stated | LC-MS | (Ferrero et al., 2011) |
| Fishing cat | *Felis vivarrarina* | Urine | Captive | PPC | (Datta and Harris, 1953) |
| Fishing cat | *Felis vivarrarina* | Urine | Not stated | LC-MS | (Ferrero et al., 2011) |
| Fossa | *Cryptoprocta ferox* | Fur / mane | Captive | GC-MS | (Vogler et al., 2007) |
| Giant panda | *Ailuropoda melanoleuca* | Anal gland sac / pouch | Wild | GC-MS | (Zhou et al., 2019) |
| Giant panda | *Ailuropoda melanoleuca* | Anogenital gland | Captive | GC-MS | (Zhang *et al.*, 2008) |
| Giant panda | *Ailuropoda melanoleuca* | Scent mark | Captive | GC-MS | (Hagey and MacDonald, 2003) |
| Giant panda | *Ailuropoda melanoleuca* | Urine | Captive | GC-MS | (Hagey and MacDonald, 2003) |
| Giant panda | *Ailuropoda melanoleuca* | Urine | Captive | SPME GC-MS | (Wilson et al., 2020) |
| Giant panda | *Ailuropoda melanoleuca* | Vagina | Captive | GC-MS | (Hagey and MacDonald, 2003) |
| Grey wolf | *Canis lupus* | Anal gland sac / pouch | Captive | GC | (Raymer et al., 1985) |
| Grey wolf | *Canis lupus* | Faeces | Wild | GC-MS | (Martín et al., 2010) |
| Grey wolf | *Canis lupus* | Urine | Captive | GC-MS | (Raymer et al., 1984) |
| Grey wolf | *Canis lupus* | Urine | Captive | GC-MS | (Raymer et al., 1986) |
| Grey wolf | *Canis lupus* | Urine | Captive | SPME GC-MS | (Osada et al., 2013) |
| Grey wolf | *Canis lupus* | Urine | Captive | SBSE | (Wolfram, 2013a) |
| Grey wolf | *Canis lupus* | Urine | Not stated | LC-MS | (Ferrero et al., 2011) |
| Hog-nosed skunk | *Conepatus leuconotus* | Anal gland sac / pouch | Wild | GC-MS | (Wood et al., 1993) |
| Hooded skunk | *Mephitis macroura* | Anal gland sac / pouch | Wild | GC-MS | (Wood et al., 2002) |
| Indian leopard cat | *Felis bengalensis* | Urine | Captive | PPC | (Datta and Harris, 1953) |
| Indian leopard cat | *Felis bengalensis* | Urine | Captive | PPC | (Hendriks et al., 1995) |
| Indian mongoose | *Herpestes auropunctatus* | Anal gland sac / pouch | Wild | GC-MS | (Miyazaki et al., 2018b) |
| Indian mongoose | *Herpestes auropunctatus* | Anal gland sac / pouch | Wild | GLC | (Gorman, 1976) |
| Jaguar | *Panthera onca* | Urine | Captive | GE-MS | (Mclean et al., 2007) |
| Jaguar | *Panthera onca* | Urine | Captive | LC-MS | (Ferrero et al., 2011) |
| Least weasel | *Mustela nivalis* | Anal gland sac / pouch | Wild | GC-MS | (Brinck et al., 1983) |
| Leopard | *Panthera pardus* | Face | Captive | SBSE | (Soini et al., 2012) |
| Leopard | *Panthera pardus* | Marking fluid | Captive | GC – TLC | (Poddar-Sarkar and Brahmachary, 2004) |
| Leopard | *Panthera pardus* | Marking fluid | Captive | TLC | (Brahmachary and Singh, 2000) |
| Leopard | *Panthera pardus* | Urine | Captive | PPC | (Datta and Harris, 1953) |
| Leopard | *Panthera pardus* | Urine | Captive | PPC | (Hendriks et al., 1995) |
| Leopard | *Panthera pardus* | Urine | Wild | GC-MS | (Apps et al., 2014) |
| Lynx (Eurasian) | *Lynx lynx* | Urine | Both | SPME GC-MS | (Vogt et al., 2016) |
| Maned wolf | *Chrysocyon brachyurus* | Urine | Captive | SPME GC-MS | (Jones, 2017a) |
| Maned wolf | *Chrysocyon brachyurus* | Urine | Captive | SPME GC-MS | (Goodwin et al., 2013) |
| Meerkat | *Suricata suricatta* | Anal gland sac / pouch | Wild | GC-MS | (Leclaire et al., 2017) |
| Meerkat | *Suricata suricatta* | Anal gland sac / pouch | Wild | GC-MS | (Leclaire et al., 2017) |
| Mountain lion / puma | *Puma concolor* | Face | Captive | SBSE | (Soini et al., 2012) |
| Mountain lion / puma | *Puma concolor* | Urine | Captive | PPC | (Datta and Harris, 1953) |
| Mountain lion / puma | *Puma concolor* | Urine | Not stated | LC-MS | (Ferrero et al., 2011) |
| Ocelot | *Leopardus pardalis* | Urine | Captive | LC-MS | (Ferrero et al., 2011) |
| Ocelot | *Leopardus pardalis* | Urine | Captive | PPC | (Datta and Harris, 1953) |
| Ocelot | *Leopardus pardalis* | Urine | Captive | PPC | (Hendriks et al., 1995) |
| Palm civet | *Paguma larvata* | Anal gland sac / pouch | Wild | GC-MS | (Wheeler et al., 1998) |
| Pardine genet | *Genetta pardina* | Anal gland sac / pouch | Captive | TLC GC-MS | (Jacob and Schliemann, 1986) |
| Persian leopard | *Panthera pardus saxicolor* | Urine | Captive | GE-MS | (Mclean et al., 2007) |
| Pinemartin | *Martes martes* | Anal gland sac / pouch | Wild | GC-MS | (Brinck et al., 1983) |
| Polecat | *Putorius putorius* | Anal gland sac / pouch | Captive | GC | (Schildknecht et al., 1976) |
| Polecat | *Putorius putorius* | Anal gland sac / pouch | Wild | GC-MS | (Brinck et al., 1983) |
| Polecat | *Putorius putorius* | Urine | Captive | PPC | (Datta and Harris, 1953) |
| Raccoon | *Procyon lotor* | Urine | Not stated | LC-MS | (Ferrero et al., 2011) |
| Red fox | *Vulpes vulpes* | Anal gland sac / pouch | Both | GC-MS | (Albone et al., 1974) |
| Red fox | *Vulpes vulpes* | Anal gland sac / pouch | Captive | GC-MS | (Albone et al., 1976) |
| Red fox | *Vulpes vulpes* | Anal gland sac / pouch | Captive | TLC GC-MS | (Albone and Gronneberg, 1977) |
| Red fox | *Vulpes vulpes* | Anal gland sac / pouch | Captive | TLC GC-MS | (Albone and Perry, 1976) |
| Red fox | *Vulpes vulpes* | Faeces | Not stated | Not given | (Vernet-Maury, 1980) |
| Red fox | *Vulpes vulpes* | Supracaudal gland | Wild | GC-MS | (McLean et al., 2019) |
| Red fox | *Vulpes vulpes* | Supracaudal gland | Wild | TLC | (Albone and Flood, 1976) |
| Red fox | *Vulpes vulpes* | Urine | Not stated | LC-MS | (Ferrero et al., 2011) |
| Red fox | *Vulpes vulpes* | Urine | Not stated | Not given | (Apps et al., 2015) |
| Red fox | *Vulpes vulpes* | Urine | Wild | GC-MS | (Jorgenson et al., 1978) |
| Red fox | *Vulpes vulpes* | Urine | Wild | GC-MS | (Wilson et al., 1978) |
| Red wolf | *Canis rufus* | Urine | Captive | SBSE | (Wolfram, 2013) |
| Ringed seal | *Phoca hispida* | Face | Wild | GC-MS | (Ryg et al., 1992) |
| Serval | *Leptailurus serval* | Urine | Captive | LC-MS | (Ferrero et al., 2011) |
| Serval | *Leptailurus serval* | Urine | Captive | PPC | (Datta and Harris, 1953) |
| Siberian tiger | *Panthera tigris altaica* | Marking fluid | Captive | GC-MS | (Soso and Koziel, 2016) |
| Siberian weasel | *Mustela sibirica* | Anal gland sac / pouch | Wild | GC-MS | (Zhang et al., 2003) |
| Siberian weasel | *Mustela sibirica* | Anal gland sac / pouch | Wild | SPME GC-MS | (Zhang et al., 2002) |
| Snow leopard | *Uncia uncia* | Marking fluid | Captive | GC-MS | (Das et al., 2019) |
| Snow leopard | *Uncia uncia* | Urine | Captive | LC-MS | (Ferrero et al., 2011) |
| Spotted hyena | *Crocuta crocuta* | Scent mark | Wild | SPME GC-MS | (Hofer et al., 2001) |
| Spotted hyena | *Crocuta crocuta* | Subcaudal gland | Wild | GC-MS | (Theis et al., 2013) |
| Spotted skunk | *Spilogale putorius* | Anal gland sac / pouch | Wild | GC-MS | (Wood et al., 1991) |
| Steppe polecat | *Mustela eversmanni* | Anal gland sac / pouch | Wild | GC-MS | (Zhang et al., 2003) |
| Steppe polecat | *Mustela eversmanni* | Anal gland sac / pouch | Wild | SPME GC-MS | (Zhang et al., 2002) |
| Stoat | *Mustela ermina* | Anal gland sac / pouch | Captive | GC | (Schildknecht et al., 1976) |
| Stoat | *Mustela ermina* | Anal gland sac / pouch | Captive | SPME GC-MS | (Burnham et al., 2008) |
| Stoat | *Mustela ermina* | Anal gland sac / pouch | Wild | GC-MS | (Brinck et al., 1983) |
| Stoat | *Mustela ermina* | Anal gland sac / pouch | Wild | GC-MS | (Crump, 1980b) |
| Stoat | *Mustela ermina* | Anal gland sac / pouch | Wild | GC-MS | (Crump and Moors, 1985) |
| Striped hyena | *Hyaena hyaena* | Anal gland sac / pouch | Not stated | GC-MS | (Wheeler et al., 1975) |
| Striped hyena | *Hyaena hyaena* | Subcaudal gland | Wild | GC-MS | (Theis et al., 2013) |
| Striped polecat | *Ictonyx striatus* | Anal gland sac / pouch | Captive | GC-MS | (Wheeler et al., 1997) |
| Striped polecat | *Ictonyx striatus* | Anal gland sac / pouch | Wild | GC-MS | (Apps et al., 1988) |
| Striped skunk | *Mephitis mephitis* | Anal gland sac / pouch | Not stated | Fractionation | (Aldrich, 1896) |
| Striped skunk | *Mephitis mephitis* | Anal gland sac / pouch | Not stated | Fractionation | (Aldrich and Jones, 1897) |
| Striped skunk | *Mephitis mephitis* | Anal gland sac / pouch | Not stated | Fractionation | (Stevens, 1945) |
| Striped skunk | *Mephitis mephitis* | Anal gland sac / pouch | Not stated | GC-MS | (Andersen and Bernstein, 1975) |
| Striped skunk | *Mephitis mephitis* | Anal gland sac / pouch | Wild | GC-MS | (Wood, 1990) |
| Striped skunk | *Mephitis mephitis* | Anal gland sac / pouch | Wild | TLC GC-MS | (Bernstein, 1979) |
| Sumatran tiger | *Panthera tigris sumatrae* | Urine | Captive | GE-MS | (Mclean et al., 2007) |
| Tiger | *Panthera tigris* | Anal gland sac / pouch | Captive | GC | (Banks et al., 1992) |
| Tiger | *Panthera tigris* | Face | Captive | SBSE | (Soini et al., 2012) |
| Tiger | *Panthera tigris* | Marking fluid | Captive | GC | (Banks et al., 1992) |
| Tiger | *Panthera tigris* | Marking fluid | Captive | GC | (Poddar-Sarkar, 1996) |
| Tiger | *Panthera tigris* | Marking fluid | Captive | GC-MS | (Burger et al., 2008) |
| Tiger | *Panthera tigris* | Marking fluid | Captive | TLC | (Brahmachary and Dutta, 1979) |
| Tiger | *Panthera tigris* | Marking fluid | Captive | TLC | (Brahmachary and Singh, 2000) |
| Tiger | *Panthera tigris altaica* | Urine | Captive | LC-MS | (Ferrero et al., 2011) |
| Tiger | *Panthera tigris* | Urine | Captive | PPC | (Brahmachary, 1996) |
| Tiger | *Panthera tigris* | Urine | Captive | PPC | (Datta and Harris, 1953) |
| Wolverine | *Gulo gulo* | Anal gland sac / pouch | Wild | GC-MS | (Wood et al., 2005) |
| Wolverine | *Gulo gulo* | Urine | Wild | GC-MS | (Wood et al., 2009) |
| Yellow mongoose | *Cynictis penicillate* | Anal gland sac / pouch | Not stated | GC-MS | (Apps et al., 1989b) |
