# Supplementary material for: Common scents? A review of potentially shared chemical signals in the order Carnivora
Source: Chem Senses. 2025 Jun 9;50:bjaf019. doi: 10.1093/chemse/bjaf019 (PMC12228039; doi:10.1093/chemse/bjaf019)
Supplement: bjaf019_suppl_Supplementary_Table_S2 [file bjaf019_suppl_supplementary_table_s2.docx]

**ESM Table 2** **Chemicals with identified putative behavioural function associations, their putative function, location, presence in sex, producing species, and citation.**

| Function | Position in animal | Chemical | Species | Latin Name | Citing publication |
| --- | --- | --- | --- | --- | --- |
| Age (juvenile status) | Anal gland sac / pouch | 2-(2-Pentenyl)furan | European otter | *Lutra lutra* | (Kean et al., 2011) |
| Age (juvenile status) | Anal gland sac / pouch | 2,9-Undecadiene | European otter | *Lutra lutra* | (Kean et al., 2011) |
| Age (juvenile status) | Anal gland sac / pouch | 2-Butylfuran | European otter | *Lutra lutra* | (Kean et al., 2011) |
| Age (juvenile status) | Anal gland sac / pouch | 2-Hexylfuran | European otter | *Lutra lutra* | (Kean et al., 2011) |
| Age (juvenile status) | Anal gland sac / pouch | 2-Octene, (E)- | European otter | *Lutra lutra* | (Kean et al., 2011) |
| Age (juvenile status) | Anal gland sac / pouch | 2-Octene, (Z)- | European otter | *Lutra lutra* | (Kean et al., 2011) |
| Age (juvenile status) | Anal gland sac / pouch | 2-Pentylfuran | European otter | *Lutra lutra* | (Kean et al., 2011) |
| Age (juvenile status) | Anal gland sac / pouch | 2-Propylfuran | European otter | *Lutra lutra* | (Kean et al., 2011) |
| Age (juvenile status) | Anal gland sac / pouch | 3-Methylbutanoic acid, ethyl ester | European otter | *Lutra lutra* | (Bradshaw et al., 2001) |
| Age (juvenile status) | Anal gland sac / pouch | 3-Octanone | European otter | *Lutra lutra* | (Kean et al., 2011) |
| Age (juvenile status) | Anal gland sac / pouch | 3-Pentylfuran | European otter | *Lutra lutra* | (Kean et al., 2011) |
| Age (juvenile status) | Anal gland sac / pouch | 5-Ethylcyclopent-1-enecarboxaldehyde | European otter | *Lutra lutra* | (Kean et al., 2011) |
| Age (juvenile status) | Anal gland sac / pouch | Cyclooctanemethanol | European otter | *Lutra lutra* | (Kean et al., 2011) |
| Age (juvenile status) | Anal gland sac / pouch | Heptanal | European otter | *Lutra lutra* | (Kean et al., 2011) |
| Age (juvenile status) | Anal gland sac / pouch | Hexanal | European otter | *Lutra lutra* | (Kean et al., 2011) |
| Age (juvenile status) | Anal gland sac / pouch | Indole | European otter | *Lutra lutra* | (Kean et al., 2011) |
| Age (juvenile status) | Faeces | Octadecadienoic acid, methyl ester | Grey wolf | *Canis lupus* | (Martín et al., 2010) |
| Age (juvenile status) | Anal gland sac / pouch | Octanal | European otter | *Lutra lutra* | (Kean et al., 2011) |
| Age (juvenile status) | Faeces | Oleic diethanolamide | Grey wolf | *Canis lupus* | (Martín et al., 2010) |
| Appeasement | Nipple | Dodecanoic acid | Dog | *Canis familiaris* | (Pageat and Gaultier, 2003) |
| Appeasement | Nipple | Dodecanoic acid | Domestic cat | *Felis domesticus* | (Pageat and Gaultier, 2003) |
| Appeasement | Nipple | Hexadecanoic acid | Dog | *Canis familiaris* | (Pageat and Gaultier, 2003) |
| Appeasement | Nipple | Hexadecanoic acid | Domestic cat | *Felis domesticus* | (Pageat and Gaultier, 2003) |
| Appeasement | Nipple | Linoleic acid | Dog | *Canis familiaris* | (Pageat and Gaultier, 2003) |
| Appeasement | Nipple | Linoleic acid | Domestic cat | *Felis domesticus* | (Pageat and Gaultier, 2003) |
| Appeasement | Nipple | Octadecanoic acid | Dog | *Canis familiaris* | (Pageat and Gaultier, 2003) |
| Appeasement | Nipple | Octadecanoic acid | Domestic cat | *Felis domesticus* | (Pageat and Gaultier, 2003) |
| Appeasement | Nipple | Oleic acid | Dog | *Canis familiaris* | (Pageat and Gaultier, 2003) |
| Appeasement | Nipple | Oleic acid | Domestic cat | *Felis domesticus* | (Pageat and Gaultier, 2003) |
| Appeasement | Nipple | Pentadecanoic acid | Dog | *Canis familiaris* | (Pageat and Gaultier, 2003) |
| Appeasement | Nipple | Tetradecanoic acid | Dog | *Canis familiaris* | (Pageat and Gaultier, 2003) |
| Appeasement | Nipple | Tetradecanoic acid | Domestic cat | *Felis domesticus* | (Pageat and Gaultier, 2003) |
| Female sex advertisement / recognition | Anal gland sac / pouch | 1-Dodecene | Meerkat | *Suricata suricatta* | (Leclaire et al., 2017) |
| Female sex advertisement / recognition | Anal gland sac / pouch | 1-Dodecene | Meerkat | *Suricata suricatta* | (Leclaire et al., 2017) |
| Female sex advertisement / recognition | Anal gland sac / pouch | 3-Ethyl-1,2-dithiolane | Ferret (domestic) | *Mustela putorius furo* | (Zhang et al., 2005a) |
| Female sex advertisement / recognition | Anogenital gland | 5-Methylhydantoin | Giant panda | *Ailuropoda melanoleuca* | (Zhang et al., 2008) |
| Female sex advertisement / recognition | Urine | Acetophenone | Grey wolf | *Canis lupus* | (Raymer et al., 1984) |
| Female sex advertisement / recognition | Anogenital gland | Erucic acid | Giant panda | *Ailuropoda melanoleuca* | (Zhang et al., 2008) |
| Female sex advertisement / recognition | Anogenital gland | Indole | Giant panda | *Ailuropoda melanoleuca* | (Zhang et al., 2008) |
| Female sex advertisement / recognition | Fur / mane | Isopropyl tetradecanoate | Fossa | *Cryptoprocta ferox* | (Vogler et al., 2007) |
| Group identity | Anal gland sac / pouch | 1,2,3,4-Tetrahydro-1,1,1-trimethylnaphthalene | European badger | *Meles meles* | (Buesching et al., 2016) |
| Group identity | Anal gland sac / pouch | 2-Chloroethylbenzene | European badger | *Meles meles* | (Buesching et al., 2016) |
| Group identity | Anal gland sac / pouch | 4-Octadecanolide | European badger | *Meles meles* | (Buesching et al., 2016) |
| Group identity | Anal gland sac / pouch | Benzaldehyde | European badger | *Meles meles* | (Buesching et al., 2016) |
| Group identity | Anal gland sac / pouch | Decanoic acid | European badger | *Meles meles* | (Buesching et al., 2016) |
| Group identity | Scent mark | Decanoic acid | Spotted hyena | *Crocuta crocuta* | (Hofer et al., 2001) |
| Group identity | Anal gland sac / pouch | Docosanoic acid methyl ester | European badger | *Meles meles* | (Buesching et al., 2016) |
| Group identity | Anal gland sac / pouch | Dodecanoic acid | European badger | *Meles meles* | (Buesching et al., 2016) |
| Group identity | Anal gland sac / pouch | Eicosanoic acid methyl ester | European badger | *Meles meles* | (Buesching et al., 2016) |
| Group identity | Anal gland sac / pouch | Hexadecanoic acid | European badger | *Meles meles* | (Buesching et al., 2016) |
| Group identity | Anal gland sac / pouch | Hexadecanoic acid, methyl ester | European badger | *Meles meles* | (Buesching et al., 2016) |
| Group identity | Anal gland sac / pouch | Hexanoic acid | European badger | *Meles meles* | (Buesching et al., 2016) |
| Group identity | Anal gland sac / pouch | Limonene | European badger | *Meles meles* | (Buesching et al., 2016) |
| Group identity | Anal gland sac / pouch | Linoleic acid | European badger | *Meles meles* | (Buesching et al., 2016) |
| Group identity | Anal gland sac / pouch | Linoleic acid, ethyl ester | European badger | *Meles meles* | (Buesching et al., 2016) |
| Group identity | Anal gland sac / pouch | Linoleic acid, methyl ester | European badger | *Meles meles* | (Buesching et al., 2016) |
| Group identity | Anal gland sac / pouch | Octadecanoic acid, ethyl ester | European badger | *Meles meles* | (Buesching et al., 2016) |
| Group identity | Anal gland sac / pouch | Octanoic acid | European badger | *Meles meles* | (Buesching et al., 2016) |
| Group identity | Anal gland sac / pouch | Oleic acid, ethyl ester | European badger | *Meles meles* | (Buesching et al., 2016) |
| Group identity | Anal gland sac / pouch | Phenylethyl alcohol | European badger | *Meles meles* | (Buesching et al., 2016) |
| Group identity | Anal gland sac / pouch | Tetracosanoic acid methyl ester | European badger | *Meles meles* | (Buesching et al., 2016) |
| Group identity | Anal gland sac / pouch | Tetradecanoic acid | European badger | *Meles meles* | (Buesching et al., 2016) |
| Group identity | Anal gland sac / pouch | Tetradecanoic acid, methyl ester | European badger | *Meles meles* | (Buesching et al., 2016) |
| Group identity | Anal gland sac / pouch | Undecanoic acid, 2-methyl- | European badger | *Meles meles* | (Buesching et al., 2016) |
| Individual advertisement / recognition | Urine | 2-Heptadecanone | Lynx (Eurasian) | *Lynx lynx* | (Vogt et al., 2016) |
| Individual advertisement / recognition | Anal gland sac / pouch | 2-Methylpropanoic acid | Domestic cat | *Felis domesticus* | (Miyazaki et al., 2018a) |
| Individual advertisement / recognition | Anal gland sac / pouch | 2-Pentylthietane | Ferret (domestic) | *Mustela putorius furo* | (Clapperton et al., 1988) |
| Individual advertisement / recognition | Anal gland sac / pouch | 2-Propylthietane | Ferret (domestic) | *Mustela putorius furo* | (Clapperton et al., 1988) |
| Individual advertisement / recognition | Urine | 2-Undecanone, 6,10-dimethyl- | Lynx (Eurasian) | *Lynx lynx* | (Vogt et al., 2016) |
| Individual advertisement / recognition | Anal gland sac / pouch | 3-Methylbutanoic acid | Domestic cat | *Felis domesticus* | (Miyazaki et al., 2018a) |
| Individual advertisement / recognition | Anal gland sac / pouch | Acetic acid | Domestic cat | *Felis domesticus* | (Miyazaki et al., 2018a) |
| Individual advertisement / recognition | Anal gland sac / pouch | Butanoic acid | Domestic cat | *Felis domesticus* | (Miyazaki et al., 2018a) |
| Individual advertisement / recognition | Anal gland sac / pouch | cis-2,3-Dimethylthietane | Ferret (domestic) | *Mustela putorius furo* | (Clapperton et al., 1988) |
| Individual advertisement / recognition | Urine | Dodecanoic acid | Lynx (Eurasian) | *Lynx lynx* | (Vogt et al., 2016) |
| Individual advertisement / recognition | Urine | Dodecanoic acid, isooctyl ester | Lynx (Eurasian) | *Lynx lynx* | (Vogt et al., 2016) |
| Individual advertisement / recognition | Anal gland sac / pouch | E-3,4-Dimethyl-1,2-dithiolane | Ferret (domestic) | *Mustela putorius furo* | (Clapperton et al., 1988) |
| Individual advertisement / recognition | Urine | Hexadecanoic acid | Lynx (Eurasian) | *Lynx lynx* | (Vogt et al., 2016) |
| Individual advertisement / recognition | Anal gland sac / pouch | Indole | Domestic cat | *Felis domesticus* | (Miyazaki et al., 2018a) |
| Individual advertisement / recognition | Anal gland sac / pouch | Indole | Ferret (domestic) | *Mustela putorius furo* | (Clapperton et al., 1988) |
| Individual advertisement / recognition | Urine | Nonanal | Lynx (Eurasian) | *Lynx lynx* | (Vogt et al., 2016) |
| Individual advertisement / recognition | Urine | Nonanoic acid | Lynx (Eurasian) | *Lynx lynx* | (Vogt et al., 2016) |
| Individual advertisement / recognition | Urine | Nonanol, 4,8-dimethyl- | Lynx (Eurasian) | *Lynx lynx* | (Vogt et al., 2016) |
| Individual advertisement / recognition | Anal gland sac / pouch | Pentanoic acid | Domestic cat | *Felis domesticus* | (Miyazaki et al., 2018a) |
| Individual advertisement / recognition | Anal gland sac / pouch | Propanoic acid | Domestic cat | *Felis domesticus* | (Miyazaki et al., 2018a) |
| Individual advertisement / recognition | Urine | Sulphur S8 | Lynx (Eurasian) | *Lynx lynx* | (Vogt et al., 2016) |
| Individual advertisement / recognition | Urine | Tetradecanoic acid | Lynx (Eurasian) | *Lynx lynx* | (Vogt et al., 2016) |
| Individual advertisement / recognition | Urine | Tetradecanoic acid, 12-methyl-, methyl ester | Lynx (Eurasian) | *Lynx lynx* | (Vogt et al., 2016) |
| Individual advertisement / recognition | Anal gland sac / pouch | trans-2,3-Dimethylthietane | Ferret (domestic) | *Mustela putorius furo* | (Clapperton et al., 1988) |
| Individual advertisement / recognition | Anal gland sac / pouch | Trimethylamine | Domestic cat | *Felis domesticus* | (Miyazaki et al., 2018a) |
| Individual advertisement / recognition | Anal gland sac / pouch | Z-3,4-Dimethyl-1,2-dithiolane | Ferret (domestic) | *Mustela putorius furo* | (Clapperton et al., 1988) |
| Male sex advertisement / recognition | Faeces | 1-Butanol | Domestic cat | *Felis domesticus* | (Uetake et al., 2018) |
| Male sex advertisement / recognition | Anal gland sac / pouch | 2,4,6,10-Tetramethylundecanoic acid | Egyptian mongoose | *Herpestes ichneumon* | (Hefetz et al., 1984) |
| Male sex advertisement / recognition | Urine | 3,5,7-Trimethyl-2-decanone | Grey wolf | *Canis lupus* | (Raymer et al., 1984) |
| Male sex advertisement / recognition | Urine | 3,5,7-Trimethyl-2-nonanone | Grey wolf | *Canis lupus* | (Raymer et al., 1984) |
| Male sex advertisement / recognition | Urine | 3,5-Dimethyl-2-octanone | Grey wolf | *Canis lupus* | (Raymer et al., 1984) |
| Male sex advertisement / recognition | Urine | 3-Isopentenyl methyl sulfide | Grey wolf | *Canis lupus* | (Raymer et al., 1984) |
| Male sex advertisement / recognition | Urine | 4-Methyl-3-heptanone | Grey wolf | *Canis lupus* | (Raymer et al., 1984) |
| Male sex advertisement / recognition | Anal gland sac / pouch | 4-Methyltridecanoic acid | Egyptian mongoose | *Herpestes ichneumon* | (Hefetz et al., 1984) |
| Male sex advertisement / recognition | Fur / mane | 6,10-Dimethyl-5,9-undecadien-2-one | Fossa | *Cryptoprocta ferox* | (Vogler et al., 2007) |
| Male sex advertisement / recognition | Anal gland sac / pouch | Dodecanoic acid, ethyl ester | Meerkat | *Suricata suricatta* | (Leclaire et al., 2017) |
| Male sex advertisement / recognition | Face | Hexadecanoic acid | Domestic cat | *Felis domesticus* | (Pageat and Gaultier, 2003) |
| Male sex advertisement / recognition | Anogenital gland | Hydroquinone | Giant panda | *Ailuropoda melanoleuca* | (Zhang et al., 2008) |
| Male sex advertisement / recognition | Urine | Methyl propyl sulfide | Grey wolf | *Canis lupus* | (Raymer et al., 1984) |
| Male sex advertisement / recognition | Face | Oleic acid | Domestic cat | *Felis domesticus* | (Pageat and Gaultier, 2003) |
| Male sex advertisement / recognition | Face | p-Hydroxyphenylacetic acid | Domestic cat | *Felis domesticus* | (Pageat and Gaultier, 2003) |
| Male sex advertisement / recognition | Face | Propanoic acid | Domestic cat | *Felis domesticus* | (Pageat and Gaultier, 2003) |
| Male sex advertisement / recognition | Anogenital gland | Squalene | Giant panda | *Ailuropoda melanoleuca* | (Zhang et al., 2008) |
| Male sex advertisement / recognition | Anal gland sac / pouch | Tetradecanoic acid, ethyl ester | Meerkat | *Suricata suricatta* | (Leclaire et al., 2017) |
| Male sex advertisement / recognition | Anal gland sac / pouch | Tetradecanoic acid, ethyl ester | Meerkat | *Suricata suricatta* | (Leclaire et al., 2017) |
| Neuter status | Urine | (E)-3-Iodo-2-octenoic acid | Maned wolf | *Chrysocyon brachyurus* | (Jones, 2017a) |
| Neuter status | Urine | ±-β,β-Dimethyl-γ-(hydroxymethyl)-γ-butyrolactone | Maned wolf | *Chrysocyon brachyurus* | (Jones, 2017a) |
| Neuter status | Urine | 1-(2-Hydroxy-5-methylphenyl)-2-buten-1-one | Maned wolf | *Chrysocyon brachyurus* | (Jones, 2017a) |
| Neuter status | Urine | 1,1'-Thiobis-cyclopentane | Maned wolf | *Chrysocyon brachyurus* | (Jones, 2017a) |
| Neuter status | Urine | 1,2-Dibutylhydrazine | Maned wolf | *Chrysocyon brachyurus* | (Jones, 2017a) |
| Neuter status | Urine | 10-Methyl-2-oxecanone | Maned wolf | *Chrysocyon brachyurus* | (Jones, 2017a) |
| Neuter status | Urine | 1-Hexanol | Maned wolf | *Chrysocyon brachyurus* | (Jones, 2017a) |
| Neuter status | Urine | 1-Methoxy-3-methyl-2-butene | Maned wolf | *Chrysocyon brachyurus* | (Jones, 2017a) |
| Neuter status | Urine | 1-Octanol | Maned wolf | *Chrysocyon brachyurus* | (Jones, 2017a) |
| Neuter status | Urine | 1-Octen-3-ol | Maned wolf | *Chrysocyon brachyurus* | (Jones, 2017a) |
| Neuter status | Urine | 2,4-Ditert-butylphenol | Maned wolf | *Chrysocyon brachyurus* | (Jones, 2017a) |
| Neuter status | Urine | 2,5-Dimethyl-3-(3-methylbutyl)pyrazine | Maned wolf | *Chrysocyon brachyurus* | (Jones, 2017a) |
| Neuter status | Urine | 2,5-Dimethyl-3-propylpyrazine | Maned wolf | *Chrysocyon brachyurus* | (Jones, 2017a) |
| Neuter status | Urine | 2,5-Dimethylpyrazine | Maned wolf | *Chrysocyon brachyurus* | (Jones, 2017a) |
| Neuter status | Urine | 2-Acetyl-3,5-dimethylpyrazine | Maned wolf | *Chrysocyon brachyurus* | (Jones, 2017a) |
| Neuter status | Urine | 2-Ethenyl-6-methylpyrazine | Maned wolf | *Chrysocyon brachyurus* | (Jones, 2017a) |
| Neuter status | Urine | 2-Methyl-6-(1-propenyl)pyrazine | Maned wolf | *Chrysocyon brachyurus* | (Jones, 2017a) |
| Neuter status | Urine | 2-Methylbut-3-en-2-ol | Maned wolf | *Chrysocyon brachyurus* | (Jones, 2017a) |
| Neuter status | Urine | 2-Methylpropanoic acid, 3-hydroxy-2,4,4-trimethylpentyl ester | Maned wolf | *Chrysocyon brachyurus* | (Jones, 2017a) |
| Neuter status | Urine | 2-Nonen-4-one | Maned wolf | *Chrysocyon brachyurus* | (Jones, 2017a) |
| Neuter status | Urine | 2-Pentylfuran | Maned wolf | *Chrysocyon brachyurus* | (Jones, 2017a) |
| Neuter status | Urine | 3,5-Dimethyl-2-propylpyrazine | Maned wolf | *Chrysocyon brachyurus* | (Jones, 2017a) |
| Neuter status | Urine | 3-Butyl-2,5-dimethylpyrazine | Maned wolf | *Chrysocyon brachyurus* | (Jones, 2017a) |
| Neuter status | Urine | 3-Ethyl-2,5-dimethylpyrazine | Maned wolf | *Chrysocyon brachyurus* | (Jones, 2017a) |
| Neuter status | Urine | 3-Ethylcyclopentanone | Maned wolf | *Chrysocyon brachyurus* | (Jones, 2017a) |
| Neuter status | Urine | 3-Hepten-2-one | Maned wolf | *Chrysocyon brachyurus* | (Jones, 2017a) |
| Neuter status | Urine | 3-Heptyne-2,6-dione, 5-methyl-5-(1-methylethyl)- | Maned wolf | *Chrysocyon brachyurus* | (Jones, 2017a) |
| Neuter status | Urine | 3-Methoxy-1-butyl acetate | Maned wolf | *Chrysocyon brachyurus* | (Jones, 2017a) |
| Neuter status | Urine | 3-Methyl-2-buten-1-ol | Maned wolf | *Chrysocyon brachyurus* | (Jones, 2017a) |
| Neuter status | Urine | 3-Methyl-3-nitrobut-1-ene | Maned wolf | *Chrysocyon brachyurus* | (Jones, 2017a) |
| Neuter status | Urine | 3-Methylbut-2-ene-1-thiol | Maned wolf | *Chrysocyon brachyurus* | (Jones, 2017a) |
| Neuter status | Urine | 3-Octen-2-one | Maned wolf | *Chrysocyon brachyurus* | (Jones, 2017a) |
| Neuter status | Urine | 3-tert-Butyl-2-pyrazolin-5-one | Maned wolf | *Chrysocyon brachyurus* | (Jones, 2017a) |
| Neuter status | Urine | 4-Ethyl-1,3-benzenediol | Maned wolf | *Chrysocyon brachyurus* | (Jones, 2017a) |
| Neuter status | Urine | 4-Methyl-2-heptanone | Maned wolf | *Chrysocyon brachyurus* | (Jones, 2017a) |
| Neuter status | Urine | Benzaldehyde | Maned wolf | *Chrysocyon brachyurus* | (Jones, 2017a) |
| Neuter status | Urine | Diphenylamine | Maned wolf | *Chrysocyon brachyurus* | (Jones, 2017a) |
| Neuter status | Urine | Isophorone | Maned wolf | *Chrysocyon brachyurus* | (Jones, 2017a) |
| Neuter status | Urine | Methyl 1-methyl-2-butenyl sulfide | Maned wolf | *Chrysocyon brachyurus* | (Jones, 2017a) |
| Neuter status | Urine | Methyl nonanoate | Maned wolf | *Chrysocyon brachyurus* | (Jones, 2017a) |
| Neuter status | Urine | N,N-Dibutylformamide | Maned wolf | *Chrysocyon brachyurus* | (Jones, 2017a) |
| Neuter status | Urine | Nonanal | Maned wolf | *Chrysocyon brachyurus* | (Jones, 2017a) |
| Neuter status | Urine | Orthoformic acid, tri-sec-butyl ester | Maned wolf | *Chrysocyon brachyurus* | (Jones, 2017a) |
| Neuter status | Urine | Phenol, 2,6-bis(1,1-dimethylethyl)-4-methyl-, methylcarbamate | Maned wolf | *Chrysocyon brachyurus* | (Jones, 2017a) |
| Neuter status | Urine | Tetrahydro-2,5-dimethyl-2H-pyranmethanol | Maned wolf | *Chrysocyon brachyurus* | (Jones, 2017a) |
| Neuter status | Urine | Tetrahydro-2-isopentyl-5-propylfuran | Maned wolf | *Chrysocyon brachyurus* | (Jones, 2017a) |
| Pair bond identity | Urine | Quinazoline | African wild dog | *Lycaon pictus* | (Jordan et al., 2016) |
| Reproductive status advertisement | Urine | 1,1'-Thiobis-cyclopentane | Maned wolf | *Chrysocyon brachyurus* | (Jones, 2017a) |
| Reproductive status advertisement | Urine | 1,1'-Thiobis-cyclopentane | Maned wolf | *Chrysocyon brachyurus* | (Jones, 2017a) |
| Reproductive status advertisement | Urine | 1,3-Di-tert-butylbenzene | Maned wolf | *Chrysocyon brachyurus* | (Jones, 2017a) |
| Reproductive status advertisement | Anal gland sac / pouch | 1,4-Octadien-3-ol | Grey wolf | *Canis lupus* | (Raymer et al., 1985) |
| Reproductive status advertisement | Urine | 1H‐Pyrrole‐2‐carboxaldehyde | Giant panda | *Ailuropoda melanoleuca* | (Wilson et al., 2020) |
| Reproductive status advertisement | Urine | 1-Octen-3-ol | Maned wolf | *Chrysocyon brachyurus* | (Jones, 2017a) |
| Reproductive status advertisement | Anal gland sac / pouch | 1-Octen-3-one | Grey wolf | *Canis lupus* | (Raymer et al., 1985) |
| Reproductive status advertisement | Urine | 2-Acetyl-6-methylpyrazine | Maned wolf | *Chrysocyon brachyurus* | (Jones, 2017a) |
| Reproductive status advertisement | Anal gland sac / pouch | 2-Decenal, (E)- | Grey wolf | *Canis lupus* | (Raymer et al., 1985) |
| Reproductive status advertisement | Anal gland sac / pouch | 2-Heptanone | Grey wolf | *Canis lupus* | (Raymer et al., 1985) |
| Reproductive status advertisement | Urine | 2-Nonen-4-one | Maned wolf | *Chrysocyon brachyurus* | (Jones, 2017a) |
| Reproductive status advertisement | Anal gland sac / pouch | 2-Nonenal | Grey wolf | *Canis lupus* | (Raymer et al., 1985) |
| Reproductive status advertisement | Anal gland sac / pouch | 2-Octan-1-ol acetate | Grey wolf | *Canis lupus* | (Raymer et al., 1985) |
| Reproductive status advertisement | Urine | 2-Octanone | Dog | *Canis familiaris* | (Dzięcioł et al., 2018) |
| Reproductive status advertisement | Anal gland sac / pouch | 2-Octenal, (E)- | Grey wolf | *Canis lupus* | (Raymer et al., 1985) |
| Reproductive status advertisement | Urine | 2-Pentanone | Dog | *Canis familiaris* | (Dzięcioł et al., 2018) |
| Reproductive status advertisement | Anal gland sac / pouch | 2-Pentylfuran | Grey wolf | *Canis lupus* | (Raymer et al., 1985) |
| Reproductive status advertisement | Anal gland sac / pouch | 3,7-Dimethyl-2-octenal | Grey wolf | *Canis lupus* | (Raymer et al., 1985) |
| Reproductive status advertisement | Urine | 3-Butyl-2,5-dimethylpyrazine | Maned wolf | *Chrysocyon brachyurus* | (Jones, 2017a) |
| Reproductive status advertisement | Urine | 3-Ethyl-2,5-dimethylpyrazine | Maned wolf | *Chrysocyon brachyurus* | (Jones, 2017a) |
| Reproductive status advertisement | Urine | 3-Hexanone | Dog | *Canis familiaris* | (Dzięcioł et al., 2018) |
| Reproductive status advertisement | Anal gland sac / pouch | 3-Methyltetrahydrothiophene | Coyote | *Canis latrans* | (Schultz et al., 1988) |
| Reproductive status advertisement | Anal gland sac / pouch | 3-Octanol | Grey wolf | *Canis lupus* | (Raymer et al., 1985) |
| Reproductive status advertisement | Anal gland sac / pouch | 3-Octanone | Grey wolf | *Canis lupus* | (Raymer et al., 1985) |
| Reproductive status advertisement | Urine | 4-Nonanone | Maned wolf | *Chrysocyon brachyurus* | (Jones, 2017a) |
| Reproductive status advertisement | Urine | Acetophenone | Dog | *Canis familiaris* | (Dzięcioł et al., 2018) |
| Reproductive status advertisement | Urine | Benzaldehyde | Dog | *Canis familiaris* | (Dzięcioł et al., 2018) |
| Reproductive status advertisement | Anal gland sac / pouch | Benzaldehyde | Grey wolf | *Canis lupus* | (Raymer et al., 1985) |
| Reproductive status advertisement | Urine | Benzaldehyde | Maned wolf | *Chrysocyon brachyurus* | (Jones, 2017a) |
| Reproductive status advertisement | Anal gland sac / pouch | Bis(3-methylbut-3-enyl) disulfide | Coyote | *Canis latrans* | (Schultz et al., 1988) |
| Reproductive status advertisement | Anal gland sac / pouch | Butanoic acid, butyl ester | Grey wolf | *Canis lupus* | (Raymer et al., 1985) |
| Reproductive status advertisement | Urine | Civetone | Giant panda | *Ailuropoda melanoleuca* | (Wilson et al., 2020) |
| Reproductive status advertisement | Urine | Decanoic acid | Giant panda | *Ailuropoda melanoleuca* | (Wilson et al., 2020) |
| Reproductive status advertisement | Urine | Dimethyl trisulfide | Dog | *Canis familiaris* | (Dzięcioł et al., 2018) |
| Reproductive status advertisement | Anal gland sac / pouch | Dodecanal | Coyote | *Canis latrans* | (Schultz et al., 1988) |
| Reproductive status advertisement | Anal gland sac / pouch | Hexanal | Grey wolf | *Canis lupus* | (Raymer et al., 1985) |
| Reproductive status advertisement | Anal gland sac / pouch | Methyl 3-methylbutyl sulfide | Coyote | *Canis latrans* | (Schultz et al., 1988) |
| Reproductive status advertisement | Urine | Methyl butyl sulfide | Dog | *Canis familiaris* | (Dzięcioł et al., 2018) |
| Reproductive status advertisement | Urine | Methyl pentyl sulfide | Dog | *Canis familiaris* | (Dzięcioł et al., 2018) |
| Reproductive status advertisement | Vagina | Methyl p-hydroxybenzoate | Dog | *Canis familiaris* | (Goodwin et al., 1979) |
| Reproductive status advertisement | Urine | Methyl propyl sulfide | Dog | *Canis familiaris* | (Dzięcioł et al., 2018) |
| Reproductive status advertisement | Urine | Methylbenzoate | Maned wolf | *Chrysocyon brachyurus* | (Jones, 2017a) |
| Reproductive status advertisement | Anal gland sac / pouch | Octanal | Coyote | *Canis latrans* | (Schultz et al., 1988) |
| Sex advertisement / recognition | Urine | 1-Undecanol | Ferret (domestic) | *Mustela putorius furo* | (Zhang et al., 2005a) |
| Sex advertisement / recognition | Urine | 2,5-Dimethylpyrazine | Ferret (domestic) | *Mustela putorius furo* | (Zhang et al., 2005a) |
| Sex advertisement / recognition | Urine | 2-Heptanone | Ferret (domestic) | *Mustela putorius furo* | (Zhang et al., 2005a) |
| Sex advertisement / recognition | Anal gland sac / pouch | 2-Methylbutanoic acid | European badger | *Meles meles* |  |
| Sex advertisement / recognition | Anal gland sac / pouch | 2-Octenal, 2-butyl- | European badger | *Meles meles* |  |
| Sex advertisement / recognition | Anal gland sac / pouch | 2-Pentylthietane | Ferret (domestic) | *Mustela putorius furo* | (Clapperton et al., 1988) |
| Sex advertisement / recognition | Anal gland sac / pouch | 2-Propylthietane | Ferret (domestic) | *Mustela putorius furo* | (Clapperton et al., 1988) |
| Sex advertisement / recognition | Urine | 3-Ethylcyclopentanone | Grey wolf | *Canis lupus* | (Raymer et al., 1986) |
| Sex advertisement / recognition | Urine | 4-Heptanone | Ferret (domestic) | *Mustela putorius furo* | (Zhang et al., 2005a) |
| Sex advertisement / recognition | Urine | Acetophenone | Grey wolf | *Canis lupus* | (Raymer et al., 1986) |
| Sex advertisement / recognition | Anal gland sac / pouch | cis-2,3-Dimethylthietane | Ferret (domestic) | *Mustela putorius furo* | (Clapperton et al., 1988) |
| Sex advertisement / recognition | Urine | Dimethoxyacetophenone | Ferret (domestic) | *Mustela putorius furo* | (Zhang et al., 2005a) |
| Sex advertisement / recognition | Anal gland sac / pouch | E-3,4-Dimethyl-1,2-dithiolane | Ferret (domestic) | *Mustela putorius furo* | (Clapperton et al., 1988) |
| Sex advertisement / recognition | Anal gland sac / pouch | Indole | Ferret (domestic) | *Mustela putorius furo* | (Clapperton et al., 1988) |
| Sex advertisement / recognition | Urine | o-Aminoacetophenone | Ferret (domestic) | *Mustela putorius furo* | (Zhang et al., 2005a) |
| Sex advertisement / recognition | Anal gland sac / pouch | Phenylethyl alcohol | Indian mongoose | *Herpestes auropunctatus* | (Miyazaki et al., 2018b) |
| Sex advertisement / recognition | Urine | Quinoline | Ferret (domestic) | *Mustela putorius furo* | (Zhang et al., 2005a) |
| Sex advertisement / recognition | Anal gland sac / pouch | trans-2,3-Dimethylthietane | Ferret (domestic) | *Mustela putorius furo* | (Clapperton et al., 1988) |
| Sex advertisement / recognition | Anal gland sac / pouch | Z-3,4-Dimethyl-1,2-dithiolane | Ferret (domestic) | *Mustela putorius furo* | (Clapperton et al., 1988) |
| Species advertisement / encoding | Urine | 2-Acetyl-1-pyrroline | Binturong | *Arctictis binturong* | (Greene et al., 2016) |
| Stimulates overmarking | Urine | 2-Phenylethyl methyl sulfide | Red fox | *Vulpes vulpes* | (Apps et al., 2015) |
| Stimulates overmarking | Urine | 3-Isopentenyl methyl sulfide | Red fox | *Vulpes vulpes* | (Apps et al., 2015) |
| Stimulates overmarking | Urine | 4-Heptanone | Red fox | *Vulpes vulpes* | (Apps et al., 2015) |
| Stimulates overmarking | Urine | 6-Methyl-5-hepten-2-one | Red fox | *Vulpes vulpes* | (Apps et al., 2015) |
| Stimulates overmarking | Urine | Acetophenone | Red fox | *Vulpes vulpes* | (Apps et al., 2015) |
| Stimulates overmarking | Urine | Benzaldehyde | Red fox | *Vulpes vulpes* | (Apps et al., 2015) |
| Stimulates overmarking | Urine | Geranyl acetone | Red fox | *Vulpes vulpes* | (Apps et al., 2015) |
| Stimulates overmarking | Urine | Quinoline, 2-methyl- | Red fox | *Vulpes vulpes* | (Apps et al., 2015) |
| Territorial marking | Marking fluid | 1,4-Diaminobutane﻿ | Leopard | *Panthera pardus* | (Poddar-Sarkar and Brahmachary, 2004) |
| Territorial marking | Marking fluid | 1,5-Diaminopentane | Leopard | *Panthera pardus* | Poddar-Sarkar and Brahmachary 2004) |
| Territorial marking | Urine | 1-Methyl-2,4-imidazolidinedione | African wild dog | *Lycaon pictus* | (Jordan et al., 2016) |
| Territorial marking | Marking fluid | 2-Acetyl-1-pyrroline | Leopard | *Panthera pardus* | Poddar-Sarkar and Brahmachary 2004) |
| Territorial marking | Urine | 2-Aminocarbonyl-1-methylimidazole | African wild dog | *Lycaon pictus* | (Jordan et al., 2016) |
| Territorial marking | Urine | 2-Methylpropanoic acid | African wild dog | *Lycaon pictus* | (Jordan et al., 2016) |
| Territorial marking | Marking fluid | 2-Methylpropanoic acid | Leopard | *Panthera pardus* | Poddar-Sarkar and Brahmachary 2004) |
| Territorial marking | Urine | 2-Phenylethyl methyl sulfide | Red fox | *Vulpes vulpes* | (Wilson et al., 1978) |
| Territorial marking | Marking fluid | 2-Phenylethylamine | Leopard | *Panthera pardus* | Poddar-Sarkar and Brahmachary 2004) |
| Territorial marking | Urine | 2-Piperidone | African wild dog | *Lycaon pictus* | (Jordan et al., 2016) |
| Territorial marking | Urine | 3-Isopentenyl methyl sulfide | Red fox | *Vulpes vulpes* | (Wilson et al., 1978) |
| Territorial marking | Marking fluid | 3-Methylbutanoic acid | Leopard | *Panthera pardus* | Poddar-Sarkar and Brahmachary 2004) |
| Territorial marking | Urine | 4-Heptanone | Red fox | *Vulpes vulpes* | (Wilson et al., 1978) |
| Territorial marking | Marking fluid | 4-Methylpentanoic acid | Leopard | *Panthera pardus* | Poddar-Sarkar and Brahmachary 2004) |
| Territorial marking | Face | 5β-Cholestan-3β-ol | Domestic cat | *Felis domesticus* | (Pageat and Gaultier, 2003) |
| Territorial marking | Urine | 6-Methyl-5-hepten-2-one | Red fox | *Vulpes vulpes* | (Wilson et al., 1978) |
| Territorial marking | Marking fluid | Acetic acid | Leopard | *Panthera pardus* | Poddar-Sarkar and Brahmachary 2004) |
| Territorial marking | Marking fluid | Acetone | Leopard | *Panthera pardus* | Poddar-Sarkar and Brahmachary 2004) |
| Territorial marking | Urine | Acetophenone | Red fox | *Vulpes vulpes* | (Wilson et al., 1978) |
| Territorial marking | Urine | Benzaldehyde | Red fox | *Vulpes vulpes* | (Wilson et al., 1978) |
| Territorial marking | Face | Butanoic acid | Domestic cat | *Felis domesticus* | (Pageat and Gaultier, 2003) |
| Territorial marking | Marking fluid | Butanoic acid | Leopard | *Panthera pardus* | Poddar-Sarkar and Brahmachary 2004) |
| Territorial marking | Marking fluid | Dimethylamine | Leopard | *Panthera pardus* | Poddar-Sarkar and Brahmachary 2004) |
| Territorial marking | Marking fluid | Ethylenediamine | Leopard | *Panthera pardus* | Poddar-Sarkar and Brahmachary 2004) |
| Territorial marking | Urine | Geranyl acetone | Red fox | *Vulpes vulpes* | (Wilson et al., 1978) |
| Territorial marking | Urine | Glycerine | African wild dog | *Lycaon pictus* | (Jordan et al., 2016) |
| Territorial marking | Marking fluid | Heptanoic acid | Leopard | *Panthera pardus* | Poddar-Sarkar and Brahmachary 2004) |
| Territorial marking | Urine | Hexadecanoic acid | African wild dog | *Lycaon pictus* | (Jordan et al., 2016) |
| Territorial marking | Face | Hexadecanoic acid | Domestic cat | *Felis domesticus* | (Pageat and Gaultier, 2003) |
| Territorial marking | Marking fluid | Hexanoic acid | Leopard | *Panthera pardus* | Poddar-Sarkar and Brahmachary 2004) |
| Territorial marking | Marking fluid | Isoheptanoic acid | Leopard | *Panthera pardus* | Poddar-Sarkar and Brahmachary 2004) |
| Territorial marking | Marking fluid | Isooctanoic acid | Leopard | *Panthera pardus* | Poddar-Sarkar and Brahmachary 2004) |
| Territorial marking | Urine | Methyl tridecanoate | African wild dog | *Lycaon pictus* | (Jordan et al., 2016) |
| Territorial marking | Urine | N,N-Dimethylurea | African wild dog | *Lycaon pictus* | (Jordan et al., 2016) |
| Territorial marking | Urine | Nonacosane | African wild dog | *Lycaon pictus* | (Jordan et al., 2016) |
| Territorial marking | Face | Nonanedioic acid | Domestic cat | *Felis domesticus* | (Pageat and Gaultier, 2003) |
| Territorial marking | Marking fluid | Nonanoic acid | Leopard | *Panthera pardus* | Poddar-Sarkar and Brahmachary 2004) |
| Territorial marking | Urine | Octadecanoic acid | African wild dog | *Lycaon pictus* | (Jordan et al., 2016) |
| Territorial marking | Marking fluid | Octanoic acid | Leopard | *Panthera pardus* | Poddar-Sarkar and Brahmachary 2004) |
| Territorial marking | Face | Oleic acid | Domestic cat | *Felis domesticus* | (Pageat and Gaultier, 2003) |
| Territorial marking | Urine | Pentadecen-1-ol | African wild dog | *Lycaon pictus* | (Jordan et al., 2016) |
| Territorial marking | Marking fluid | Pentanoic acid | Leopard | *Panthera pardus* | Poddar-Sarkar and Brahmachary 2004) |
| Territorial marking | Face | Pimelic acid | Domestic cat | *Felis domesticus* | (Pageat and Gaultier, 2003) |
| Territorial marking | Marking fluid | Propanoic acid | Leopard | *Panthera pardus* | Poddar-Sarkar and Brahmachary 2004) |
| Territorial marking | Urine | Quinazoline | African wild dog | *Lycaon pictus* | (Jordan et al., 2016) |
| Territorial marking | Urine | Quinoline, 2-methyl- | Red fox | *Vulpes vulpes* | (Wilson et al., 1978) |
| Territorial marking | Urine | Tetradecanoic acid | African wild dog | *Lycaon pictus* | (Jordan et al., 2016) |
| Territorial marking | Marking fluid | Trimethylamine | Leopard | *Panthera pardus* | Poddar-Sarkar and Brahmachary 2004) |
| Time of year /  season | Anal gland sac / pouch | (E)-7-Tetradecene | Giant panda | *Ailuropoda melanoleuca* | (Zhou et al., 2019) |
| Time of year /  season | Anal gland sac / pouch | 1-Docosanol | Giant panda | *Ailuropoda melanoleuca* | (Zhou et al., 2019) |
| Time of year /  season | Anal gland sac / pouch | 1-Hexadecanol | Giant panda | *Ailuropoda melanoleuca* | (Zhou et al., 2019) |
| Time of year /  season | Anal gland sac / pouch | 1-Octanol | Giant panda | *Ailuropoda melanoleuca* | (Zhou et al., 2019) |
| Time of year /  season | Anal gland sac / pouch | 2,5-Hexanedione | Giant panda | *Ailuropoda melanoleuca* | (Zhou et al., 2019) |
| Time of year /  season | Anal gland sac / pouch | 2-Heptenal | Giant panda | *Ailuropoda melanoleuca* | (Zhou et al., 2019) |
| Time of year /  season | Anal gland sac / pouch | 2-Octanone | Giant panda | *Ailuropoda melanoleuca* | (Zhou et al., 2019) |
| Time of year /  season | Anal gland sac / pouch | 2-Undecanone | Giant panda | *Ailuropoda melanoleuca* | (Zhou et al., 2019) |
| Time of year /  season | Urine | 3-Isopentenyl methyl sulfide | Grey wolf | *Canis lupus* | (Raymer et al., 1984) |
| Time of year /  season | Anal gland sac / pouch | 6,10,14-Trimethyl-2-pentadecanone | Giant panda | *Ailuropoda melanoleuca* | (Zhou et al., 2019) |
| Time of year /  season | Fur / mane | 6,10-Dimethyl-5,9-undecadien-2-one | Fossa | *Cryptoprocta ferox* | (Vogler et al., 2007) |
| Time of year /  season | Anal gland sac / pouch | Cholesta-5,7-dien-3-ol acetate | Giant panda | *Ailuropoda melanoleuca* | (Zhou et al., 2019) |
| Time of year /  season | Anal gland sac / pouch | Dodecanal | Giant panda | *Ailuropoda melanoleuca* | (Zhou et al., 2019) |
| Time of year /  season | Anal gland sac / pouch | Heneicosanone | Giant panda | *Ailuropoda melanoleuca* | (Zhou et al., 2019) |
| Time of year /  season | Fur / mane | Isopropyl tetradecanoate | Fossa | *Cryptoprocta ferox* | (Vogler et al., 2007) |
| Time of year /  season | Anal gland sac / pouch | Tetracosanol | Giant panda | *Ailuropoda melanoleuca* | (Zhou et al., 2019) |
| Time of year /  season | Anal gland sac / pouch | Tricosanone | Giant panda | *Ailuropoda melanoleuca* | (Zhou et al., 2019) |
